# Supplementary material for: “Ion-imprinting” strategy towards metal sulfide scavenger enables the highly selective capture of radiocesium
Source: Nat Commun. 2024 May 20;15:4281. doi: 10.1038/s41467-024-48565-x (PMC11106286; doi:10.1038/s41467-024-48565-x)
Supplement: Supplementary file 1 — Supplementary Information [file 41467_2024_48565_MOESM1_ESM.pdf]

“Ion-imprinting” strategy towards metal sulfide scavenger enables the highly selective capture of radiocesium

Jun-Hao Tang[1,2], Shao-Qing Jia[3], Jia-Ting Liu[1], Lu Yang[1], Hai-Yan Sun[1], Mei-Ling Feng*[,1,2,4] and Xiao-Ying Huang[1,2]

[1] State Key Laboratory of Structural Chemistry, Fujian Institute of Research on the Structure of Matter, Chinese Academy of Sciences, Fuzhou, Fujian 350002, P. R. China

[2] University of Chinese Academy of Sciences, Beijing, 100049, P. R. China

[3] HTA Co., Ltd., Beijing, 102413, P. R. China

[4] Fujian Province Joint Innovation Key Laboratory of Fuel and Materials in Clean Nuclear Energy System, Fujian Institute of Research on the Structure of Matter, Chinese Academy of Sciences Fuzhou, 350002, P. R. China

*Corresponding author. Email: fml@fjirsm.ac.cn

Supplementary Notes 1. Equation.

(1) Removal rate (R):

$$R = \frac{(C_0 - C_\mathrm{e})}{C_\mathrm{e}} \times 100\% \qquad \text{(Eq. 1)}$$

where C_0 (mg L^{-1}) and C_e (mg L^{-1}) are the initial and equilibrium concentration of target ions, respectively.

(2) Distribution coefficient (K_d):

$$K_\mathrm{d} = \frac{V}{m}\frac{(C_0 - C_\mathrm{e})}{C_\mathrm{e}} \qquad \text{(Eq. 2)}$$

(3) Kinetics model[1]:

Pseudo-first-order kinetics model:

$$\ln(q_\mathrm{e} - q_\mathrm{t}) = \ln q_\mathrm{e} - k_1 t \qquad \text{(Eq. 3)}$$

Pseudo-second-order kinetics model:

$$\frac{t}{q_t} = \frac{1}{k_2 q_\mathrm{e}^2} + \frac{t}{q_\mathrm{e}} \qquad \text{(Eq. 4)}$$

where q_e (mg g^{-1}) and q_t (mg g^{-1}) are the ion exchange capacities at equilibrium and time t (min), respectively. k_1 (min^{-1}) and k_2 (g mg^{-1} min^{-1}) are pseudo-first-order and pseudo-second-order rate constants of kinetics models, respectively.

(4) Adsorption isotherm models[2]:

$$q_e = q_\mathrm{m}\frac{bC_\mathrm{e}}{1 + bC_\mathrm{e}} \qquad \text{(Eq. 5)}$$

$$q_e = K_\mathrm{F} C_e^{1/n} \qquad \text{(Eq. 6)}$$

$$q_e = q_\mathrm{m}\frac{(bC_\mathrm{e})^{1/n}}{(1 + bC_\mathrm{e})^{1/n}} \qquad \text{(Eq. 7)}$$

where C_e (mg L^{-1}) is the concentration at adsorption equilibrium, q_e (mg g^{-1}) is the amount of cation adsorbed per unit of adsorbent at adsorption equilibrium, q_m is the maximum adsorption capacity of the adsorbent, and b (L mg^{-1}) is the Langmuir constant which is relevant to the free energy of exchange. K_F and n is the Freundlich constant.

Where C_0 (mg L^{-1}) and C_e (mg L^{-1}) are the initial and equilibrium concentrations of the target ion, respectively; m (g) and V (mL) are the mass of the ion exchange material and the volume of the solution used in the ion exchange experiment, respectively.

(5) Leaching rate (R_L):

$$R_\mathrm{L} = \frac{C_\mathrm{P}}{C_\mathrm{T}} \times 100\% \qquad \text{(Eq. 8)}$$

where R_L(%) is the leaching rate, C_P (mg L^{-1}) is the concentration of Ga or Sn ions in the solution after soaking the samples, and C_T (ppm) is the theoretical concentration of Ga or Sn ions when the sample is completely dissolved in the solution.

(6):

Considering the loss of solids due to washing and drying, as well as the change in material weight due to the exchange of Cs^+ ions with NH_4^+ ions, the corresponding data need to be corrected when calculating the desorption rate (Here, the effects caused by the entry and exit of water molecules into and out of the structure are ignored.). The desorption rate can be considered as the percentage of the mass of Cs^+ ions desorbed off to the mass of Cs^+ ions adsorbed on. Taking into account the loss of solid mass, it can be calculated according to the following formula:

$$E = \frac{C_e^{de} V_{de}}{m_2 (m_{Cs}^{ad} / m_{ad})} \times 100\% \qquad \text{(Eq. 9)}$$

where C_e^{de} (mg L^{-1}) is the Cs^+ concentration in the solution after desorption. V_{de} (L) and m_2 (mg) are the volume of solution and mass of solid sample used in the desorption process, respectively. m_{Cs}^{ad} (mg) is the mass of adsorbed Cs^+ ion during the adsorption process. m_{ad} (mg) is the mass of the solid sample after adsorption of Cs^+. Mass loss during washing and drying results in the solid sample obtained from the adsorption process not being fully utilized in the desorption process, and therefore m_2 is not equivalent to m_{ad}. Therefore, m_2 needs to be weighed to determine while m_{ad} needs to be calculated using the correction formula Eq. 11. In Eq. 9, m_{Cs}^{ad}/m_{ad} represents the ratio of the mass of adsorbed Cs^+ ions to the total mass of adsorbent at the end of the adsorption process. m_{Cs}^{ad} can be calculated using the following equation:

$$m_{Cs}^{ad} = \left(C_0^{ad} - C_e^{ad}\right) V_{ad} \qquad \text{(Eq. 10)}$$

where C_0^{ad} (mg L^{-1}) and C_e^{ad} (mg L^{-1}) are the initial and adsorption equilibrium concentrations of Cs^+ ions in solution during the adsorption process, respectively. V_{ad} (L) is the volume of the solution added during the adsorption. In addition, considering that the exchange of Cs^+ with NH_4^+ makes the total weight of the exchange product not equal to the mass of the added material, it is necessary to correct m_{ad} by the following equation:

$$m_{ad} = m_1 + m_{Cs}^{ad} - M_{NH4^+} \times m_{Cs}^{ad} / M_{Cs^+} \qquad \text{(Eq. 11)}$$

where $M_{Cs^+} = 132.9$ and $M_{NH4^+} = 18$. Eq. 11 means that the mass of the solid at the end of the adsorption process (m_{ad}) is equal to the mass of the adsorbent added (m_1) plus the mass of the adsorbed Cs^+ ions (m_{Cs}^{ad}) and minus the mass of the NH_4^+ ions exchanged out ($M_{NH4^+} \times m_{Cs}^{ad}/M_{Cs^+}$).

(7) Thomas models[3-4]:

$$\frac{C_t}{C_0} = \frac{1}{1 + \exp(K_T q_e m / Q - K_T C_0 t)} \qquad \text{(Eq. 12)}$$

where C_0 (mg L^{-1}) and C_t (mg L^{-1}) are the concentrations at the initial and time t (min), respectively. K_T (L min^{-1} mg^{-1}) is the rate constant in the Thomas model. q_e (mg g^{-1}) is the maximum adsorption capacity of the adsorbent, Q (L min^{-1}) is the volume flow rate, and m (g) is the mass of the adsorbent in the exchange column.

(8) Elution rate (R_E):

$$R_E = \frac{m_E}{m_{total}} \times 100\% \qquad \text{(Eq. 13)}$$

where m_E is the mass of eluted Cs^+ ions over a period of time and m_{total} is total mass of eluted Cs^+ ions.

(8) Binding energy:

$$E_{ads} = E_{total} - (E_{GTS} + E_{ions}) \qquad \text{(Eq. 14)}$$

where E_{ads} is the adsorptive binding energies. E_{total} is the total energy of the interaction system between the $[Ga_{2.33}Sn_{1.67}S_8]_n^{2.33n-}$ layer and metal ion. The E_{CTS} and E_{ions} are the energies of the $[Ga_{2.33}Sn_{1.67}S_8]_n^{2.33n-}$ layer and the isolated ions, respectively.

Supplementary Method. Additional notes on some of the characterization and experiments.

2.1 Kinetic Study of Activating FJSM-CGTS. In order to investigate the optimal time for the activation of FJSM-CGTS by 2 mol L^{-1} KCl to obtain FJSM-KCGTS, kinetic experiments of the activation process were carried out. The degree of the sample being activated was reflected by testing the concentration of Cs^+ in the solution. It was shown that the concentration of Cs^+ ions in the solution basically reached equilibrium at about 360 min (Supplementary Fig. 10). To ensure sufficient activation, the activation time of the FJSM-KCGTS samples used for ion exchange testing were all set at 24 hours.

2.2 XPS analysis of FJSM-CGTS, FJSM-KCGTS, and FJSM-KCGTS-Cs. The appearance of K $2p_{3/2}$ and K $2p_{1/2}$ peaks located at 292.1 eV and 294.8 eV and the attenuation of Cs $3d_{5/2}$ and Cs $3d_{3/2}$ peaks located at 723.3 eV and 737.2 eV were observed in the XPS plots of FJSM-KCGTS (Supplementary Fig. 5), which confirms that most of Cs^+ were exchanged by K^+ ions. The attenuation of the K 2*p*3/2 and K 2*p*1/2 peaks and the enhancement of the Cs $3d_{5/2}$ and Cs $3d_{3/2}$ peaks were found in the XPS spectra of FJSM-KCGTS-Cs (Supplementary Fig. 5). It is confirmed that most of K^+ in FJSM-KCGTS were exchanged by Cs^+.

2.3 Adsorption-elution cycle experiment. In order to investigate the reuse ability of the materials, we performed adsorption-desorption cycle experiments. The results showed that R^{Cs} remained above 98.97% after three cycles, and the desorption rates (Eq. 9) were all around 100% (Supplementary Fig. 14). Note that the desorption rate of more than 100% in the first cycle was attributed to the small amount of Cs^+ remaining in the initial material being further eluted at this point. The experimental results indicate that the adsorbed Cs^+ can be completely desorbed in each cycle and the desorbed material can still be used again for Cs^+ capture with high efficiency.

2.4 Actual Water Sampling Experiments. Cs^+ was added to lake, river and seawater to simulate contaminated water bodies. Then the Cs^+ removal ability of FJSM-KCGTS is investigated in environmental water bodies. In the river and lake water samples, R^{Cs} reached 98.30% and 97.60%, respectively, with K_d^{Cs} both higher than 10^4 mL g^{-1}. R^{Cs} of FJSM-KCGTS could reach 51.78% in seawater samples with high salinity when the adsorbent was added at a dosage of 1 g L^{-1} (Supplementary Fig. 19a). In addition, we examined the ability of FJSM-KCGTS to capture Cs^+ in seawater solutions containing different concentrations of Cs^+ ions. The adsorption isotherm data in seawater were better fitted to the Langmuir-Freundlich isotherm model (R^2 = 0.99) with q_m^{Cs} of 206 mg g^{-1} (Supplementary Fig. 19b, Supplementary Table 8). Although seawater will not actually contain so much Cs^+, the experimental results can still confirm that FJSM-KCGTS can selectively capture Cs^+ in so complex solutions containing a large number of competing ions.

2.5 Comparison with other materials

Three-dimensional microporous metal sulfide ion exchangers with specific pore sizes have shown excellent Cs^+ trapping properties[5-9]. In order to compare the selectivity of FJSM-KGCTS with these materials under the same experimental conditions, we synthesized 3D-K_6MS as well as 3D-$[(Me)_2NH_2]_{0.75}[Ag_{1.25}SnSe_3]$ and performed their Cs^+ selective capture experiments. In addition, we also synthesized FJSM-GAS-1 as a representative two-dimensional material. The synthesized samples were confirmed as pure phase by PXRD test (Supplementary Fig. 20).

The results shown in Supplementary Fig. 21a demonstrate that in seawater with high salinity and complex environment, 3D-$[(Me)_2NH_2]_{0.75}[Ag_{1.25}SnSe_3]$ and 2D-FJSM-KCGTS can effectively capture Cs^+, whereas the Cs^+ capture performance of the 3D-K_6MS material and 2D-FJSM-GAS-1 is greatly affected. 3D-K_6MS and 3D-$[(Me)_2NH_2]_{0.75}[Ag_{1.25}SnSe_3]$ hardly adsorb Sr^{2+} and Eu^{3+} ions in Sr/Cs or Eu/Cs solutions. However, the presence of competing ions also made the R^{Cs} values of 3D-K_6MS and 3D-$[(Me)_2NH_2]_{0.75}[Ag_{1.25}SnSe_3]$ be less than 60% and 75%, respectively (Supplementary Fig. 21b-c), even though the initial concentration of Cs^+ was only about 5 mg L^{-1}. By contrast, 2D-FJSM-KCGTS maintained a high R^{Cs} value (Supplementary Fig. 21b-c). Even in the presence of high concentrations of competing ions, it can efficiently remove low concentrations of Cs^+ in solutions. In Sr/Cs and Eu/Cs solutions, the R^{Cs} values of 2D-FJSM-KCGTS can reach 98.98% and 98.90%, respectively. By contrast, the R^{Cs} range of FJSM-GAS-1 in Sr/Cs and Eu/Cs solutions is 7.89-51.82% and 1.37-34.57%, respectively, and the R^{Cs} is less than 1%

in seawater samples. Therefore, compared with 2D-FJSM-GAS-1, 2D-FJSM-KCGTS has significantly better performance for selective capture of Cs^+.

2.6 Column Experiments. The concentration of all elution samples collected during the elution phase was measured. The last sample had a Cs^+ concentration of about 0.2219 mg L^{-1}, which is approximated as complete elution. The total mass of Cs^+ eluted was the sum of the mass of Cs^+ in each sample tube (Total: m_e^{Cs} = 33.555 mg). The first four eluent samples (totaling 12 mL) eluted 18.64 mg of Cs^+ ions, accounting for 55.55% of the total mass of Cs^+ ions eluted.

Supplementary Notes 2. Results of the characterization and experiments.

Supplementary Table 1. Crystallographic data and structural refinements for compounds FJSM-CGTS, FJSM-KCGTS, and FJSM-KCGTS-Cs.

Compounds	FJSM-CGTS	FJSM-KCGTS	FJSM-KCGTS-Cs
Empirical formula	$Cs_{2.33}Ga_{2.33}Sn_{1.67}S_8H_2O$	$K_{1.82}Cs_{0.51}Ga_{2.33}Sn_{1.67}S_8H_2O$	$Cs_{2.12}K_{0.21}Ga_{2.33}Sn_{1.67}S_8H_2O$
Formula weight	945.12	774.07	925.64
Temperature/K	297(2)	294(2)	297(2)
Wavelength/Å	0.71073	0.71073	0.71073
Crystal system	Orthorhombic	Orthorhombic	Orthorhombic
space group	$Pmc2_1$	$Pmc2_1$	$Pmc2_1$
a/Å	7.3869(10)	7.3510(14)	7.4419(17)
b/Å	10.1502(13)	9.960(2)	10.1895(18)
c/Å	12.2135(18)	11.865(2)	12.251(2)
V/ Å^3	915.8(2)	868.7(3)	929.0(3)
Z	2	2	2
$D_{calc.}$ (mg m^{-3})	3.428	2.959	3.309
F (000)	844	713	829
Crystal size/mm^3	0.10 × 0.10 × 0.01	0.15 × 0.15 × 0.01	0.15 × 0.15 × 0.01
θ range for data collection/ °	3.411 to 29.353	3.434 to 29.679	3.776 to 30.121
Limiting indices	-9<=h<=10, -13<=k<=11, -15<=l<=12	-10<=h<=8, -12<=k<=11, -15<=l<=14	-9<=h<=10, -13<=k<=11, -17<=l<=14
Completeness to θ = 25.242 °	98.5%	99.6%	99.2%
Refinement method	Full-matrix least-squares on F^2	Full-matrix least-squares on F^2	Full-matrix least-squares on F^2
Data / restraints / parameters	1745 / 14 / 108	1980 / 7 / 100	2292 / 13 / 97
Goodness-of-fit on F^2	1.083	1.078	1.090
Final R indices [I > 2σ(I)]	R_1 = 0.0569, wR_2 = 0.1320	R_1 = 0.0486, wR_2 = 0.1146	R_1 = 0.0545, wR_2 = 0.1157
R indices (all data)	R_1 = 0.0698, wR_2 = 0.1412	R_1 = 0.0567, wR_2 = 0.1201	R_1 = 0.0759, wR_2 = 0.1336
CCDC	2288484	2288487	2312868

[a] $R_1 = \sum||F_o| - |F_c||/\sum|F_o|$, $wR_2 = [\sum w(F_o^2-F_c^2)^2/\sum w(F_o^2)^2]^{1/2}$.

Supplementary Table 2. Selected bond lengths (Å) and angles (°) for compound FJSM-CGTS.

M(1)-S(5)#1	2.328(5)	S(1)-Cs(1)#4	3.525(7)	S(5)-Cs(2B)#7	3.578(12)
M(1)-S(5)	2.328(5)	S(2)-Cs(3)#8	3.532(10)	S(5)-Cs(3B)	3.78(5)
M(1)-S(4)	2.329(7)	S(2)-Cs(2B)	3.576(11)	S(5)-Cs(2)	3.784(10)
M(1)-S(6)#2	2.374(8)	S(2)-Cs(1)#4	3.888(5)	S(5)-Cs(2)#7	3.841(9)
M(2)-S(3)	2.323(4)	S(2)-Cs(3B)#8	4.02(4)	S(5)-Cs(3)	3.855(17)
M(2)-S(2)	2.326(6)	S(2)-Cs(2)	4.022(9)	S(5)-Cs(1)	3.929(6)
M(2)-S(5)	2.328(5)	S(3)-Cs(3)#9	3.47(2)	S(5)-Cs(2B)	4.064(13)
M(2)-S(1)	2.344(4)	S(3)-Cs(3B)#9	3.61(5)	S(6)-Cs(1)	3.516(7)
M(3)-S(2)#5	2.295(5)	S(4)-Cs(1)#2	3.590(8)	S(6)-Cs(2)#5	3.7083(11)
M(3)-S(2)#6	2.295(5)	S(4)-Cs(3)	3.6947(7)	S(6)-Cs(2)#7	3.7083(11)
M(3)-S(4)	2.301(8)	S(4)-Cs(3)#3	3.6947(7)	S(6)-Cs(2B)#7	3.7209(19)
M(3)-S(6)	2.302(7)	S(4)-Cs(3B)	3.729(6)	S(6)-Cs(2B)#5	3.7209(19)
S(1)-Cs(1)	3.428(8)	S(4)-Cs(3B)#3	3.729(6)		

S(5)#1-*M*(1)-S(5)	113.3(3)	S(2)-*M*(2)-S(5)	114.7(2)	S(2)#6-*M*(3)-S(6)	112.14(18)
S(5)#1-*M*(1)-S(4)	112.27(16)	S(3)-*M*(2)-S(1)	104.63(19)	S(4)-*M*(3)-S(6)	106.2(3)
S(5)-*M*(1)-S(4)	112.27(16)	S(2)-*M*(2)-S(1)	110.7(2)	*M*(2)-S(1)-*M*(2)#1	104.8(2)
S(5)#1-*M*(1)-S(6)#2	110.53(18)	S(5)-*M*(2)-S(1)	110.9(2)	*M*(3)#2-S(2)-*M*(2)	103.1(2)
S(5)-*M*(1)-S(6)#2	110.53(18)	S(2)#5-*M*(3)-S(2)#6	113.9(3)	*M*(2)-S(3)-*M*(2)#10	104.4(3)
S(4)-*M*(1)-S(6)#2	96.8(3)	S(2)#5-*M*(3)-S(4)	105.86(19)	*M*(3)-S(4)-*M*(1)	110.9(3)
S(3)-*M*(2)-S(2)	109.1(2)	S(2)#6-*M*(3)-S(4)	105.86(19)	*M*(2)-S(5)-*M*(1)	103.6(2)
S(3)-*M*(2)-S(5)	106.3(2)	S(2)#5-*M*(3)-S(6)	112.14(18)	*M*(3)-S(6)-*M*(1)#5	105.5(3)

(*M*(1) = 0.433 Ga + 0.567 Sn; *M*(2) = 0.589 Ga + 0.411 Sn; *M*(3) = 0.72 Ga + 0. 28 Sn)

Symmetry transformations used to generate equivalent atoms: #1 -*x*+1, *y*, *z*; #2 -*x*+1, -*y*+1, *z*-1/2; #3 *x*+1, *y*, *z*; #4 -*x*+1, -*y*, *z*-1/2; #5 -*x*+1, -*y*+1, *z*+1/2; #6 *x*, -*y*+1, *z*+1/2; #7 -*x*, -*y*+1, *z*+1/2; #8 -*x*, -*y*+1, *z*-1/2; #9 *x*, *y*-1, *z*; #10 -x, y, z.

Supplementary Table 3. Selected bond lengths (Å) and angles (°) for compound FJSM-KCGTS.

M(1)-S(4)	2.310(6)	S(1)-K(1)	3.256(8)	S(4)-K(3B)#3	3.787(19)
M(1)-S(5)	2.328(4)	S(1)-K(1)#7	3.488(9)	S(5)-K(2)	3.41(3)
M(1)-S(5)#1	2.328(4)	S(2)-K(3B)#8	3.31(6)	S(5)-K(3B)	3.35(6)
M(1)-S(6)#2	2.361(6)	S(2)-K(1)#7	3.731(7)	S(5)-Cs(2)#6	3.625(5)
M(2)-S(2)	2.309(4)	S(2)-K(3)#8	3.71(3)	S(5)-K(3)	3.79(3)
M(2)-S(3)	2.314(3)	S(2)-Cs(2)	3.884(9)	S(5)-Cs(2)	3.787(6)
M(2)-S(5)	2.324(4)	S(3)-K(3)#9	3.35(4)	S(6)-K(1)	3.484(9)
M(2)-S(1)	2.335(3)	S(4)-K(1)#2	3.357(8)	S(6)-Cs(2)#6	3.6793(9)
M(3)-S(4)	2.283(6)	S(4)-K(3)	3.6788(19)	S(6)-Cs(2)#4	3.6793(9)
M(3)-S(2)#4	2.286(4)	S(4)-K(3)#3	3.679(2)	S(6)-K(2)#4	3.711(8)
M(3)-S(2)#5	2.286(4)	S(4)-K(3B)	3.787(19)	S(6)-K(2)#6	3.711(8)
M(3)-S(6)	2.299(6)				
S(4)-*M*(1)-S(5)	112.52(12)	S(3)-*M*(2)-S(5)	106.07(17)	S(2)#4-*M*(3)-S(6)	112.19(13)
S(4)-*M*(1)-S(5)#1	112.52(12)	S(2)-*M*(2)-S(1)	110.83(18)	S(2)#5-*M*(3)-S(6)	112.19(13)
S(5)-*M*(1)-S(5)#1	113.4(2)	S(3)-*M*(2)-S(1)	104.48(15)	*M*(2)-S(1)-*M*(2)#1	105.3(2)
S(4)-*M*(1)-S(6)#2	95.2(2)	S(5)-*M*(2)-S(1)	109.83(18)	*M*(3)#2-S(2)-*M*(2)	103.54(16)
S(5)-*M*(1)-S(6)#2	110.95(13)	S(4)-*M*(3)-S(2)#4	106.91(14)	*M*(2)-S(3)-*M*(2)#10	103.6(2)
S(5)#1-*M*(1)-S(6)#2	110.94(13)	S(4)-*M*(3)-S(2)#5	106.91(14)	*M*(3)-S(4)-*M*(1)	108.5(2)
S(2)-*M*(2)-S(3)	109.58(17)	S(2)#4-*M*(3)-S(2)#5	113.1(2)	*M*(2)-S(5)-*M*(1)	103.01(16)
S(2)-*M*(2)-S(5)	115.39(17)	S(4)-*M*(3)-S(6)	104.9(2)	*M*(3)-S(6)-*M*(1)#4	105.5(2)

(*M*(1) = 0.433 Ga + 0.567 Sn; *M*(2) = 0.59 Ga + 0.41 Sn; *M*(3) = 0.72 Ga + 0.28 Sn)

Symmetry transformations used to generate equivalent atoms: #1 -*x*+1, *y*, *z*; #2 -*x*+1, -*y*+1, *z*-1/2; #3 *x*+1, *y*, *z*; #4 -*x*+1, -*y*+1, *z*+1/2; #5 *x*, -*y*+1, *z*+1/2; #6 -*x*, -*y*+1, *z*+1/2; #7 -*x*+1, -*y*, *z*-1/2; #8 -*x*, -*y*+1, *z*-1/2; #9 *x*, *y*-1, *z*; #10 -x, y, z.

Supplementary Table 4. Selected bond lengths (Å) and angles (°) for compound FJSM-KCGTS -Cs.

M(1)-S(4)	2.338(7)	S(1)-Cs(1)#5	3.538(6)	S(5)-Cs(3B)	3.83(5)
M(1)-S(5)	2.356(5)	S(2)-K(2)	3.53(6)	S(5)-Cs(3)	3.835(10)
M(1)-S(5)#1	2.356(5)	S(2)-Cs(3)#3	3.660(10)	S(5)-Cs(3B)#3	3.84(5)
M(1)-S(6)#2	2.372(6)	S(2)-Cs(2)	3.881(11)	S(5)-Cs(2)	3.883(11)
M(2)-S(3)	2.334(4)	S(2)-Cs(1)#5	3.899(5)	S(5)-Cs(1)	3.925(6)
M(2)-S(2)	2.335(5)	S(2)-Cs(3B)#3	4.13(5)	S(6)-Cs(1)	3.521(7)
M(2)-S(5)	2.336(5)	S(3)-Cs(3)#9	3.525(12)	S(6)-Cs(2)#8	3.7234(10)
M(2)-S(1)	2.362(4)	S(4)-Cs(1)#2	3.600(7)	S(6)-Cs(2)#6	3.7234(10)
M(3)-S(2)#6	2.303(5)	S(4)-Cs(3)	3.7219(9)	S(6)-Cs(3B)#4	3.743(6)
M(3)-S(2)#7	2.303(5)	S(4)-Cs(3)#4	3.7219(9)	S(6)-Cs(3B)	3.743(6)
M(3)-S(4)	2.302(7)	S(5)-K(2)#8	3.47(5)	S(6)-K(2)#8	3.789(14)
M(3)-S(6)	2.323(7)	S(5)-Cs(2)#8	3.760(11)	S(6)-K(2)#6	3.789(14)
S(1)-Cs(1)	3.442(6)				
S(4)-*M*(1)-S(5)	112.27(15)	S(2)-*M*(2)-S(5)	115.12(18)	S(2)#7-*M*(3)-S(6)	112.08(16)
S(4)-*M*(1)-S(5)#1	112.27(15)	S(3)-*M*(2)-S(1)	104.79(17)	S(4)-*M*(3)-S(6)	106.3(2)
S(5)-*M*(1)-S(5)#1	112.8(3)	S(2)-*M*(2)-S(1)	110.3(2)	*M*(2)-S(1)-*M*(2)#1	104.8(2)
S(4)-*M*(1)-S(6)#2	96.6(2)	S(5)-*M*(2)-S(1)	110.7(2)	*M*(3)#2-S(2)-*M*(2)	103.54(19)
S(5)-*M*(1)-S(6)#2	110.92(15)	S(2)#6-*M*(3)-S(2)#7	113.4(3)	*M*(2)-S(3)-*M*(2)#10	104.7(2)
S(5)#1-*M*(1)-S(6)#2	110.91(15)	S(2)#6-*M*(3)-S(4)	106.15(16)	*M*(3)-S(4)-*M*(1)	111.0(3)
S(3)-*M*(2)-S(2)	109.1(2)	S(2)#7-*M*(3)-S(4)	106.15(16)	*M*(2)-S(5)-*M*(1)	103.32(19)
S(3)-*M*(2)-S(5)	106.2(2)	S(2)#6-*M*(3)-S(6)	112.08(16)	*M*(3)-S(6)-*M*(1)#6	105.6(3)

(*M*(1) = 0.433 Ga + 0.567 Sn; *M*(2) = 0.59 Ga + 0.41 Sn; *M*(3) = 0.72 Ga + 0.28 Sn)

Symmetry transformations used to generate equivalent atoms: #1 -*x*+1, *y*, *z*; #2 -*x*+1, -*y*+1, *z*-1/2; #3 -*x*, -*y*+1, *z*-1/2; #4 *x*+1, *y*, *z*; #5 -*x*+1, -*y*, *z*-1/2; #6 -*x*+1, -*y*+1, *z*+1/2; #7 *x*, -*y*+1, *z*+1/2; #8 -*x*, -*y*+1, *z*+1/2; #9 *x*, *y*-1, *z*; #10 -*x*, *y*, *z*.

Supplementary Table 5. Atomic coordinates (× 10^4), equivalent isotropic displacement parameters (Å^2 × 10^3), SOFs and atomic sites for FJSM-CGTS, FJSM-KCGTS, and FJSM-KCGTS-Cs. *U*(eq) is defined as one third of the trace of the orthogonalized U^{ij} tensor.

Compounds	Atom	*x*	*y*	*z*	*U*(eq)	SOF	site
FJSM-CGTS	Sn(1)	5000	4650(2)	5167(2)	22(1)	0.567	2b
	Ga(1)	5000	4650(2)	5167(2)	22(1)	0.433	2b
	Sn(2)	2486(2)	1573(2)	4811(2)	23(1)	0.411	4c
	Ga(2)	2486(2)	1573(2)	4811(2)	23(1)	0.589	4c
	Sn(3)	5000	7138(2)	7505(2)	25(1)	0.28	2b
	Ga(3)	5000	7138(2)	7505(2)	25(1)	0.72	2b
	S(1)	5000	340(6)	5378(6)	23(1)	1	2b
	S(2)	2397(6)	1730(5)	2912(5)	31(1)	1	4c
	S(3)	0	368(6)	5409(6)	30(2)	1	2a
	S(4)	5000	6875(7)	5634(6)	33(2)	1	2b
	S(5)	2367(6)	3583(5)	5726(5)	31(1)	1	4c
	S(6)	5000	5049(7)	8239(6)	31(2)	1	2b
	Cs(1)	5000	1598(2)	7983(2)	64(1)	0.95	2b
	Cs(2)	0	5248(8)	3353(7)	63(2)	0.58	2a
	Cs(2B)	0	4790(11)	2895(10)	64(3)	0.384	2a
	Cs(3)	0	6966(18)	5657(11)	64(3)	0.32	2a
	Cs(3B)	0	6820(50)	5220(40)	65(9)	0.1	2a
	O(1)	0	8370(60)	3120(40)	170(20)	1	2a
FJSM-KCGTS	Sn(1)	5000	4664(2)	5138(1)	23(1)	0.5666	2b
	Ga(1)	5000	4664(2)	5138(1)	23(1)	0.4334	2b
	Sn(2)	2474(2)	1541(1)	4830(1)	22(1)	0.41	4c
	Ga(2)	2474(2)	1541(1)	4830(1)	22(1)	0.59	4c
	Sn(3)	5000	7215(2)	7439(2)	25(1)	0.28	2b
	Ga(3)	5000	7215(2)	7439(2)	25(1)	0.72	2b
	S(1)	5000	328(5)	5454(5)	28(1)	1	2b
	S(2)	2406(5)	1638(4)	2885(3)	34(1)	1	4c
	S(3)	0	328(5)	5475(4)	27(1)	1	2a
	S(4)	5000	6937(6)	5529(4)	40(2)	1	2b
	S(5)	2354(5)	3602(4)	5744(4)	35(1)	1	4c
	S(6)	5000	5073(6)	8161(4)	36(1)	1	2b
	K(1)	5000	1580(7)	7988(5)	94(3)	1	2b
	Cs(2)	0	5090(9)	3195(5)	80(2)	0.51	2a
	K(2)	0	5030(60)	3580(40)	80(2)	0.3	2a
	K(3)	0	6970(30)	5400(30)	114(10)	0.37	2a
	K(3B)	0	6430(70)	6170(60)	100(20)	0.1534	2a
	O(1W)	770(80)	8330(40)	3180(30)	140(30)	0.5	4c

Compounds	Atom	*x*	*y*	*z*	*U*(eq)	SOF	site
FJSM-KCGTS-Cs	Sn(1)	5000	4647(2)	5141(2)	22(1)	0.5666	2b
	Ga(1)	5000	4647(2)	5141(2)	22(1)	0.4334	2b
	Sn(2)	2484(2)	1567(1)	4789(1)	21(1)	0.41	4c
	Ga(2)	2484(2)	1567(1)	4789(1)	21(1)	0.59	4c
	Sn(3)	5000	7142(2)	7475(2)	22(1)	0.28	2b
	Ga(3)	5000	7142(2)	7475(2)	22(1)	0.72	2b
	S(1)	5000	328(5)	5357(5)	22(1)	1	2b
	S(2)	2414(6)	1721(5)	2888(4)	30(1)	1	4c
	S(3)	0	362(6)	5379(5)	26(1)	1	2a
	S(4)	5000	6872(7)	5609(5)	33(2)	1	2b
	S(5)	2363(7)	3569(5)	5715(4)	29(1)	1	4c
	S(6)	5000	5046(6)	8222(5)	29(2)	1	2b
	Cs(1)	5000	1605(2)	7958(2)	72(1)	1	2b
	Cs(2)	0	5079(11)	3183(11)	66(4)	0.6	2a
	K(2)	0	4700(70)	2680(50)	66(4)	0.21	2a
	Cs(3)	0	6908(9)	5548(9)	81(3)	0.323(7)	2a
	Cs(3B)	0	4670(50)	8340(50)	81(3)	0.201(7)	2a
	O(1W)	490(60)	8380(30)	3080(30)	83(13)	0.5	4c

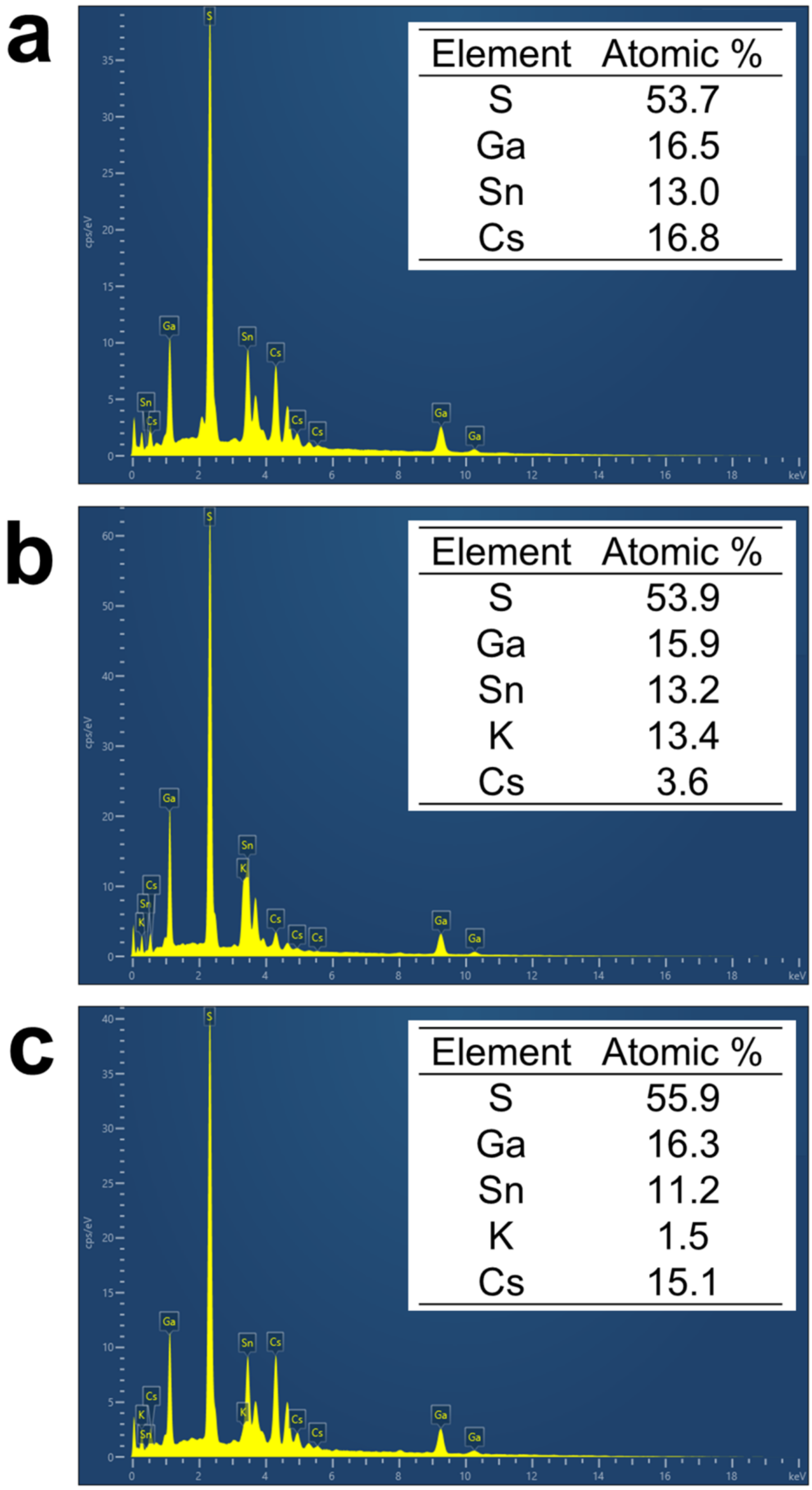

Supplementary Fig. 1. EDS analysis of FJSM-CGTS and its exchange products. EDS analysis results of (a) FJSM-CGTS, (b) FJSM-KCGTS, and (c) FJSM-KCGTS-Cs.

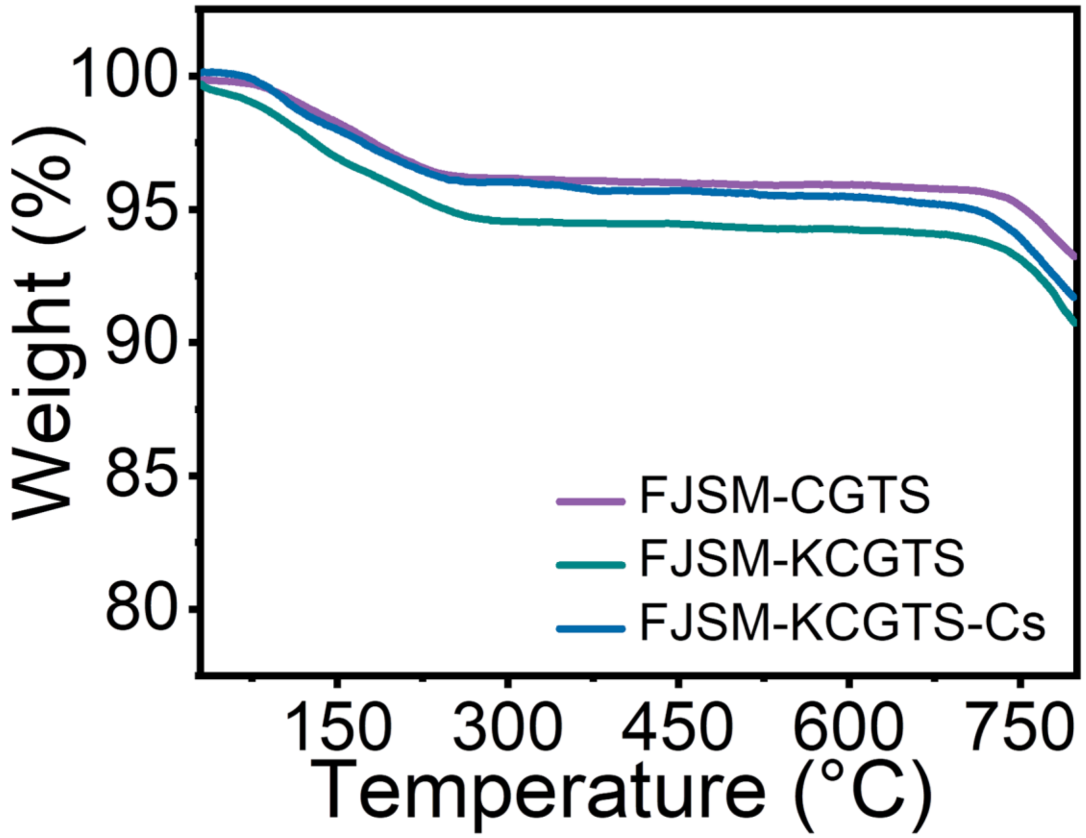

Supplementary Fig. 2. TG curves of FJSM-CGTS, FJSM-KCGTS, and FJSM-KCGTS-Cs. Source data are provided as a Source Data file.

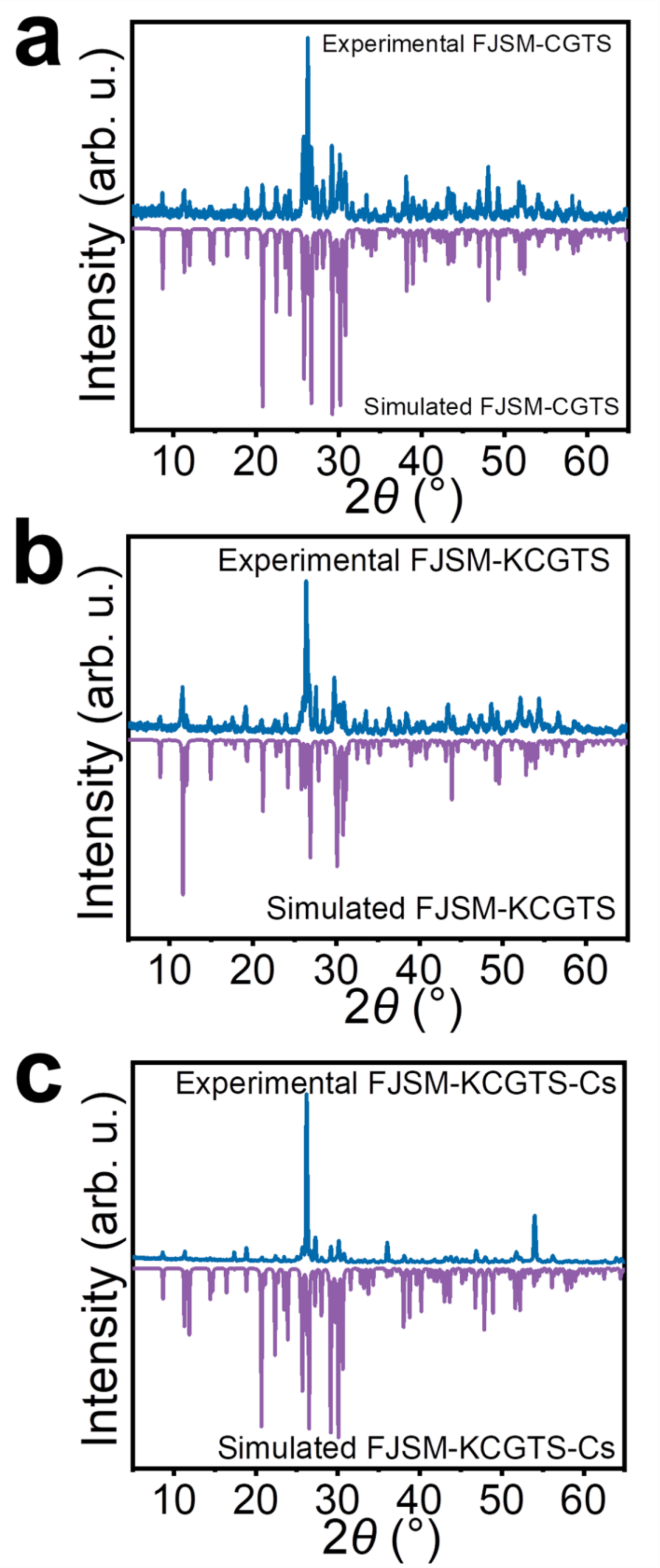

Supplementary Fig. 3. Comparison of experimental and simulated PXRD patterns of FJSM-CGTS and its exchange products. Experimental and simulated PXRD patterns of (a) FJSM-CGTS, (b) FJSM-KCGTS, and (c) FJSM-KCGTS-Cs. Source data are provided as a Source Data file.

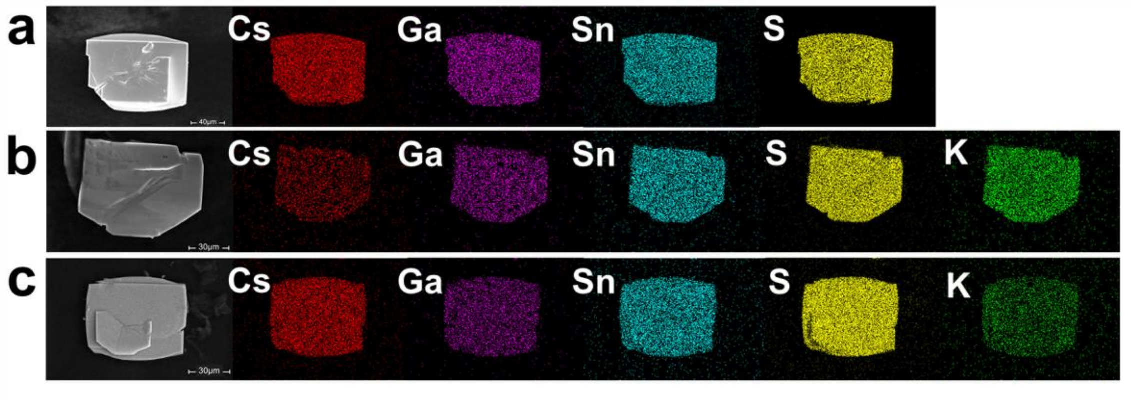

Supplementary Fig. 4. Element distribution analysis of FJSM-CGTS and its exchange products. Elemental distribution maps of (a) FJSM-CGTS, (b) FJSM-KCGTS, and (c) FJSM-KCGTS-Cs.

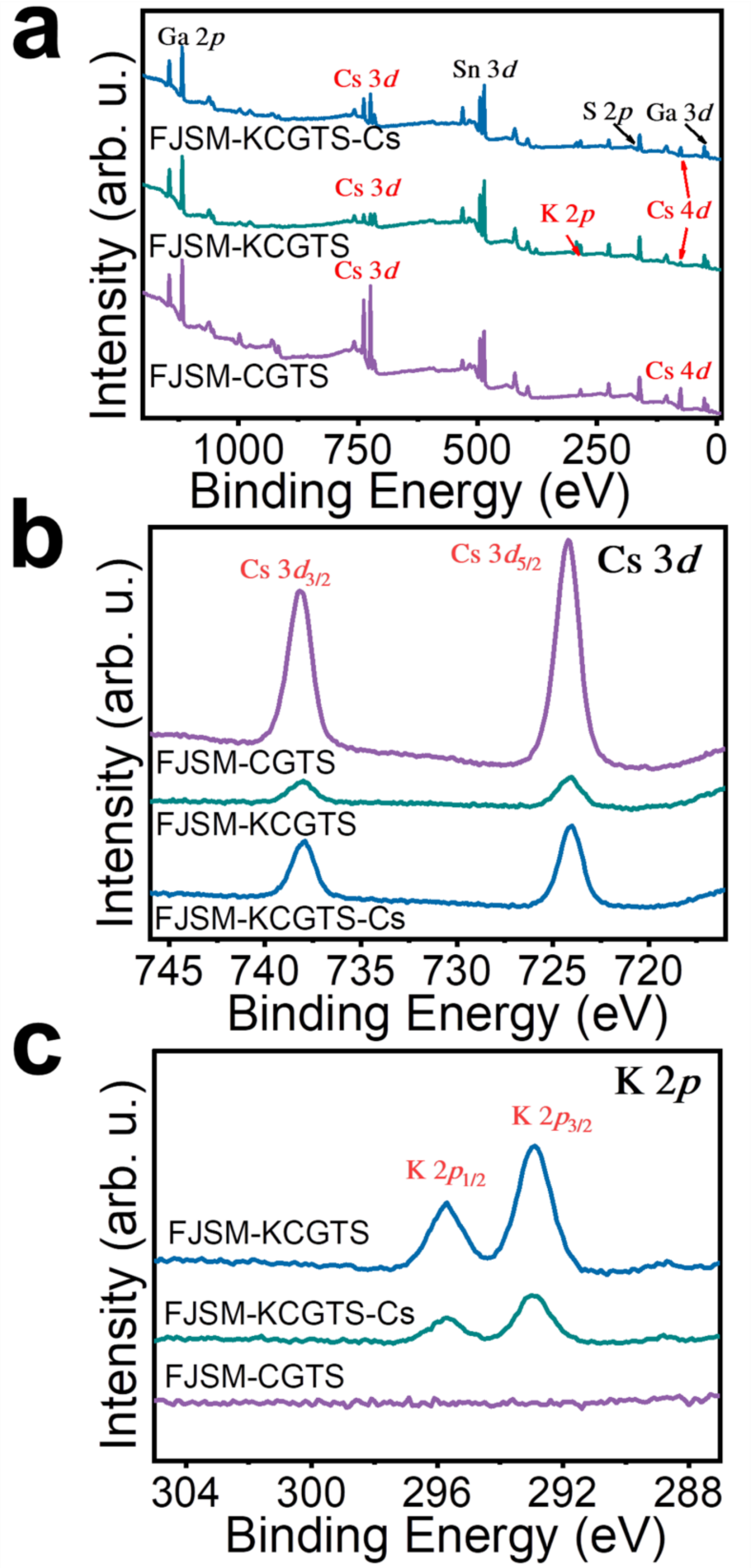

Supplementary Fig. 5. XPS analysis of FJSM-CGTS and its exchange products. (a) X-ray photoelectron spectra of FJSM-CGTS, FJSM-KCGTS, and FJSM-KCGTS-Cs; narrow scan XPS spectra of Cs 3d (b) and K 2p (c) of FJSM-CGTS, FJSM-KCGTS, and FJSM-KCGTS-Cs. Source data are provided as a Source Data file.

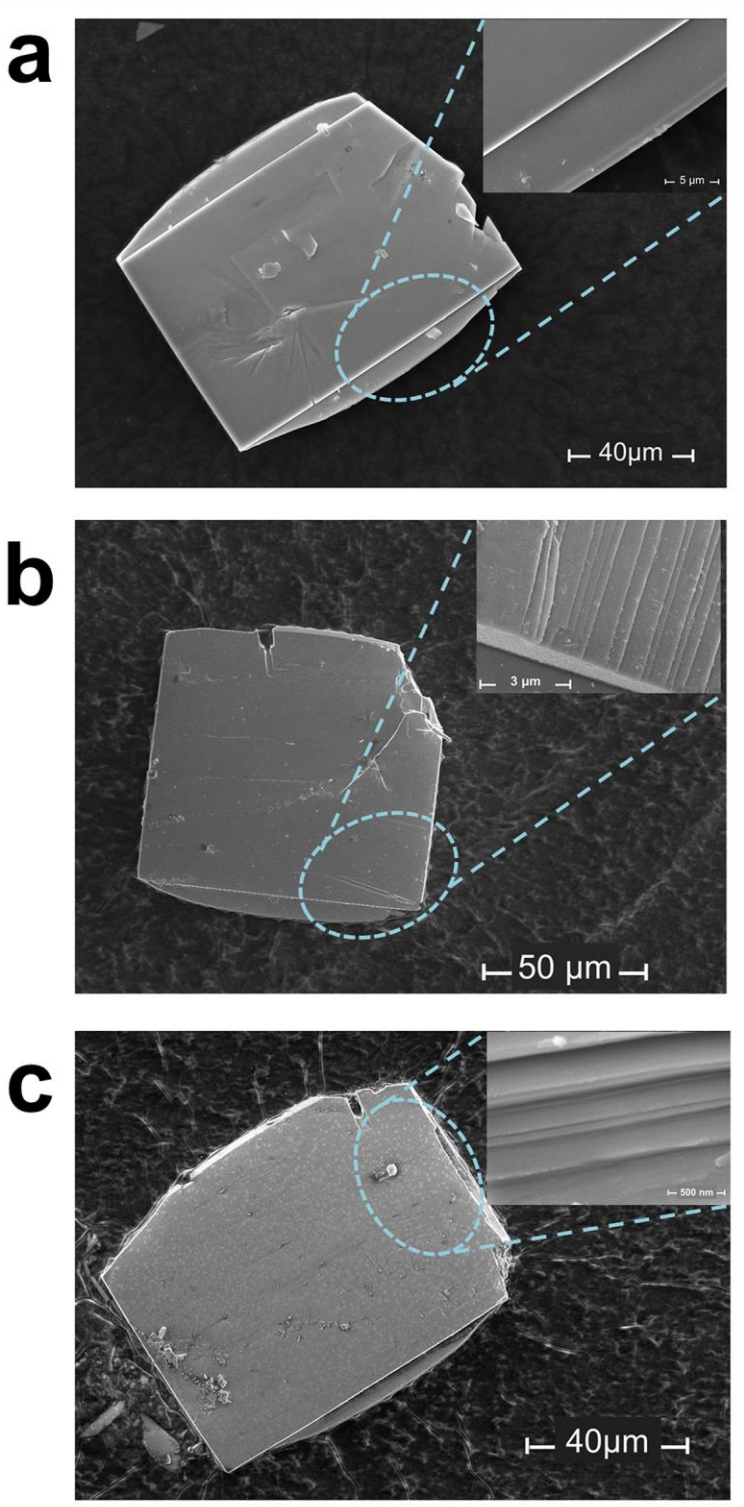

Supplementary Fig. 6. Morphology images of FJSM-CGTS and its exchange products. SEM images of (a) FJSM-CGTS, (b) FJSM-KCGTS, and (c) FJSM-KCGTS-Cs. Insets are partial enlargement images of the corresponding samples. It can be seen that FJSM-CGTS, FJSM-KCGTS, and FJSM-KCGTS-Cs have obvious layered structures. After K^+ activation and Cs^+ adsorption, the surface of the material is smooth and complete, and the morphology is not obviously broken.

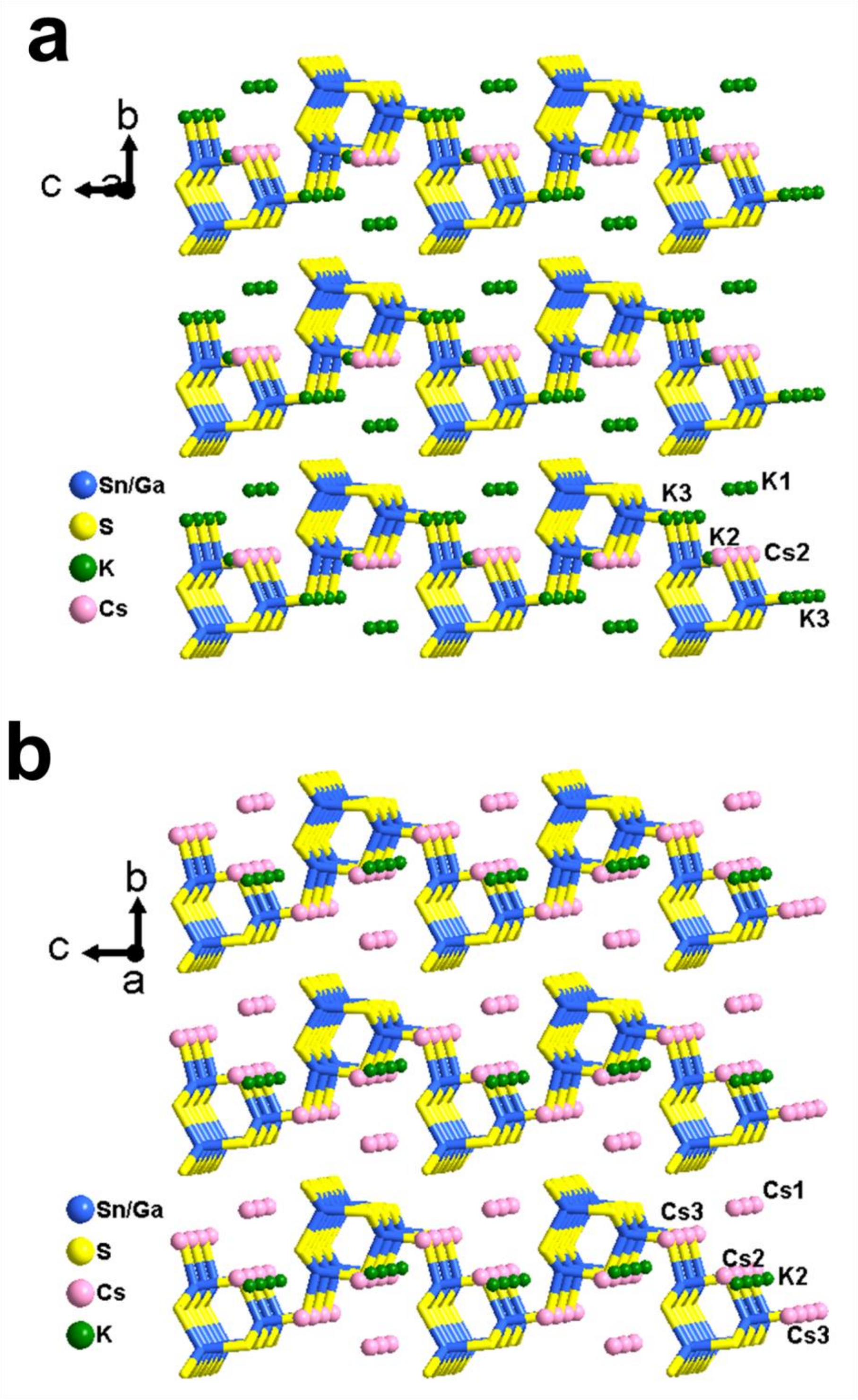

Supplementary Fig. 7. Structures of FJSM-KCGTS and FJSM-KCGTS-Cs. View of layers stacking in (a) FJSM-KCGTS and (b) FJSM-KCGTS-Cs along the *a* axis. K3B, Cs3B, O, and H are ignored for clarity.

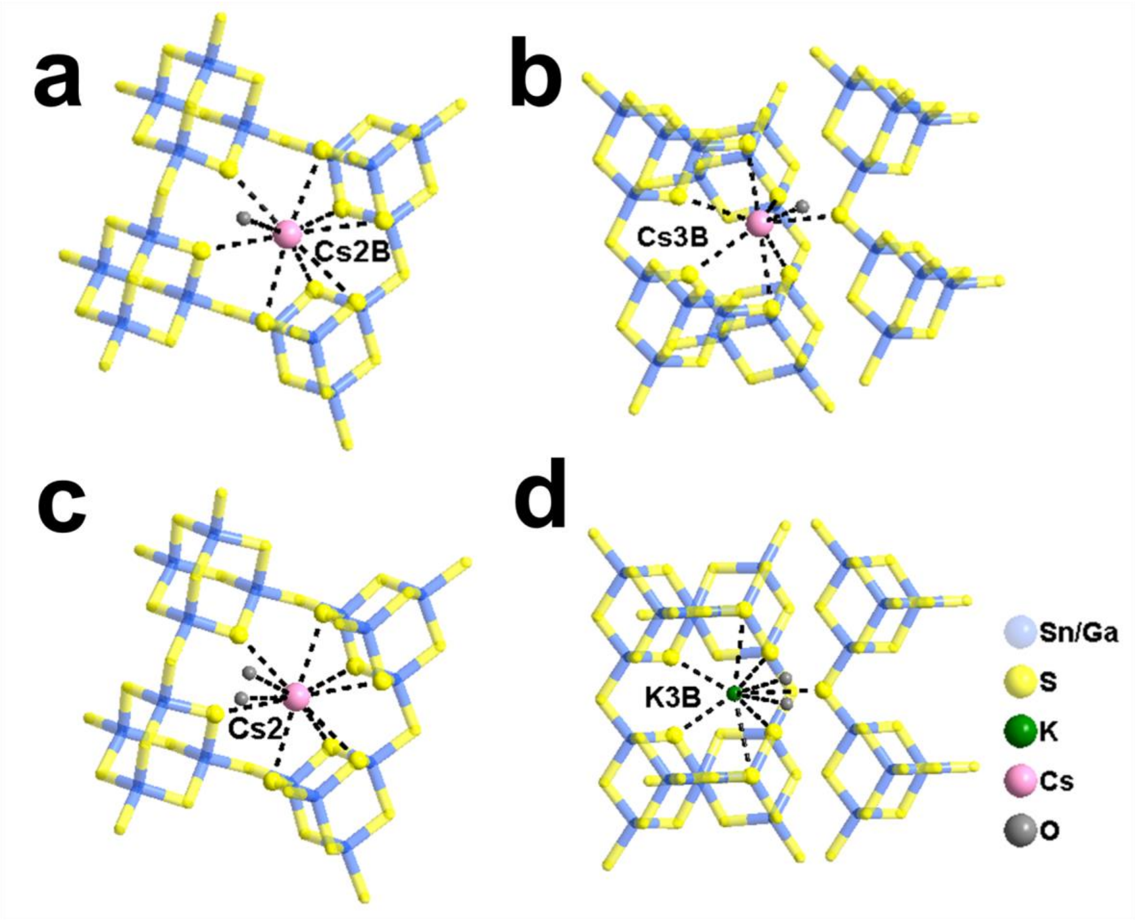

Supplementary Fig. 8. Local structure of FJSM-CGTS and FJSM-KCGTS. Coordination patterns of (a) Cs2B, (b) Cs3B in FJSM-CGTS and (c) Cs2, (d) K3B in FJSM-KCGTS. For clarity, only S or O atoms coordinated to the corresponding Cs^+ are highlighted.

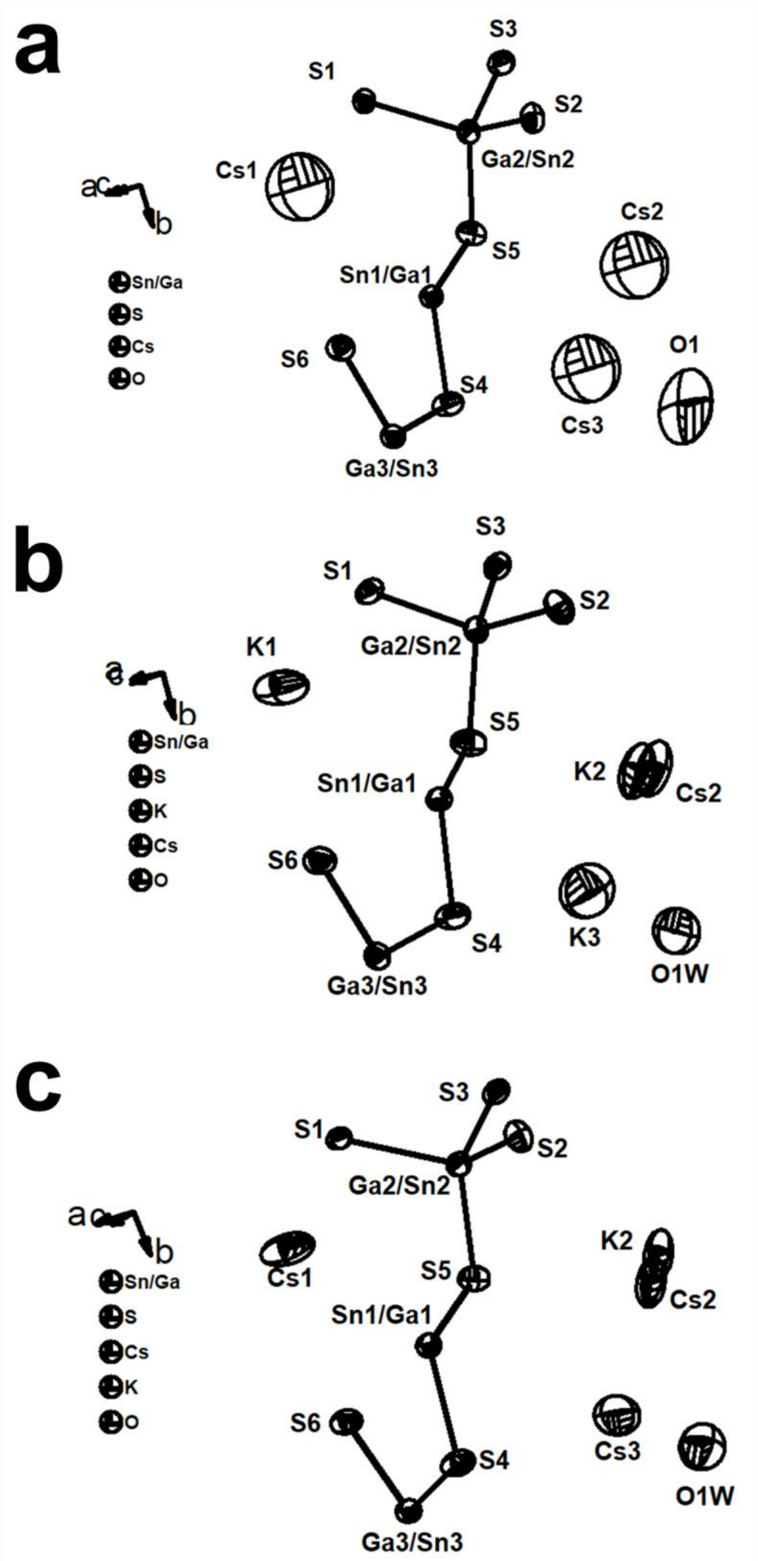

Supplementary Fig. 9. *ORTEP* drawings of FJSM-CGTS and its exchange products. *ORTEP* drawings (50% ellipsoid probability) of the non-hydrogen atoms in asymmetric units of (a) FJSM-CGTS, (b) FJSM-KCGTS, and (c) FJSM-KCGTS-Cs.

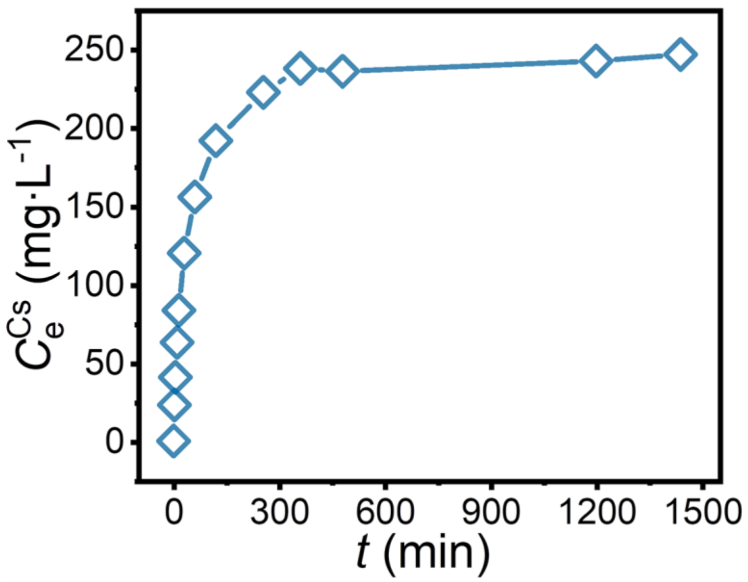

Supplementary Fig. 10. Kinetics of activation process for FJSM-CGTS plotted as Cs^+ concentration *vs* time *t* (min). The blue line is artificially added to reflect the variation trend of the Cs^+ concentration. Source data are provided as a Source Data file.

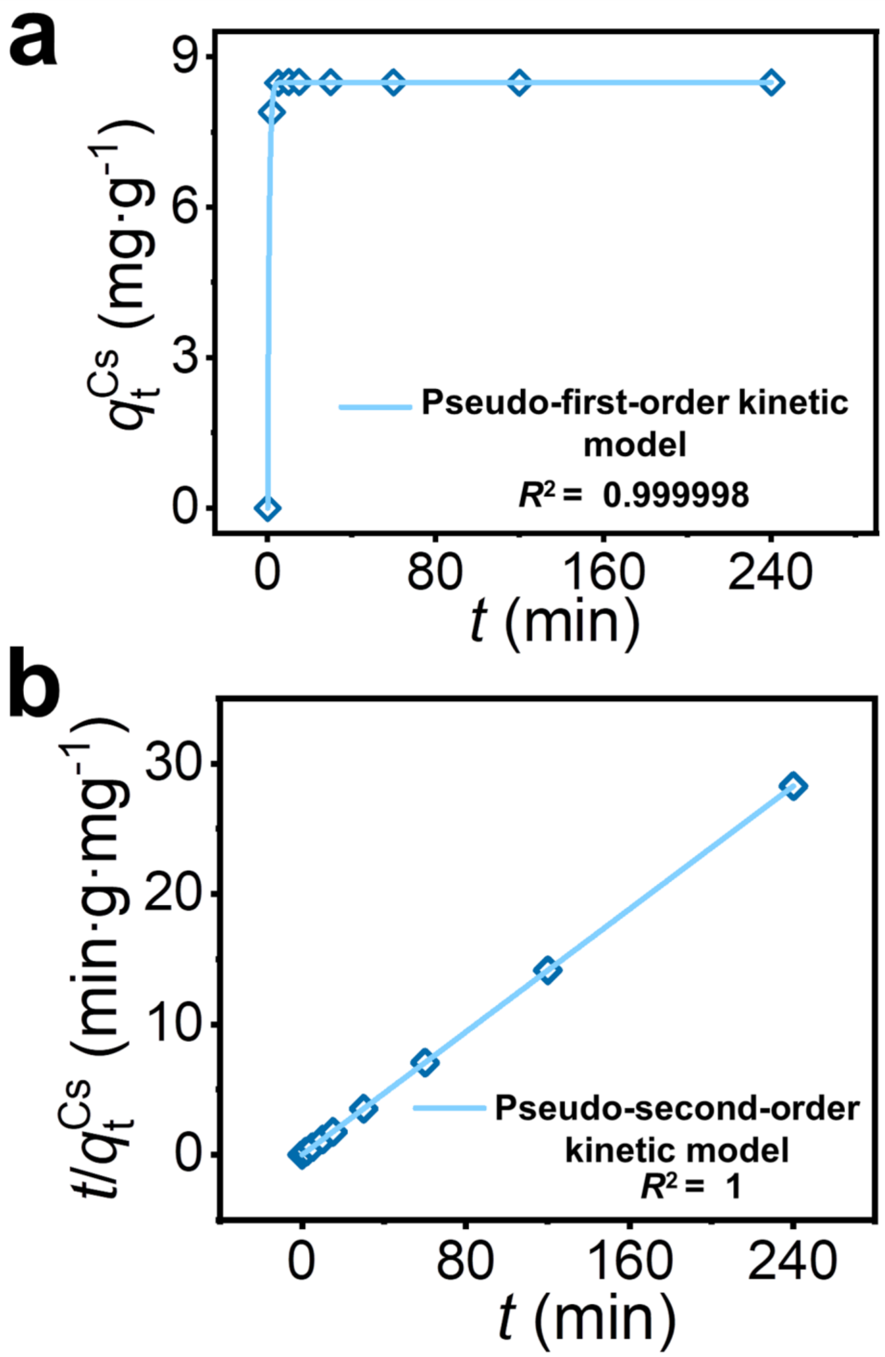

Supplementary Fig. 11. Kinetic model fitting curves for kinetic data of Cs^+ ions adsorption. (a) Pseudo-first-order kinetic model and (b) pseudo-second-order kinetic model fitting curves for kinetic data of Cs^+ ions capture by FJSM-KCGTS.

Supplementary Table 6. Kinetic fitting parameters for the capture of Cs^+ ions by FJSM-KCGTS.

Pseudo-first-order model			Pseudo-second-order model		
k_1 (min^{-1})	q_e (mg g^{-1})	R^2	k_2 (g mg^{-1} min^{-1})	q_e (mg g^{-1})	R^2
1.32759	8.49049	0.999998	5.55263	8.48752	1

Supplementary Table 7. Isotherm fitting parameters for Cs^+ capture by FJSM-KCGTS.

Langmuir model			
q_m (mg g^{-1})	b (L mg^{-1})	R^2	
245.56	1.0922	0.98819	
Freundlich model			
K_F [(mg g^{-1})(L mg^{-1})$^{1/n}$]	n	R^2	
81.166	5.0418	0.90050	
Langmuir-Freundlich model			
q_m (mg g^{-1})	b (L mg^{-1})	n	R^2
246.65	1.0453	1.0692	0.98847

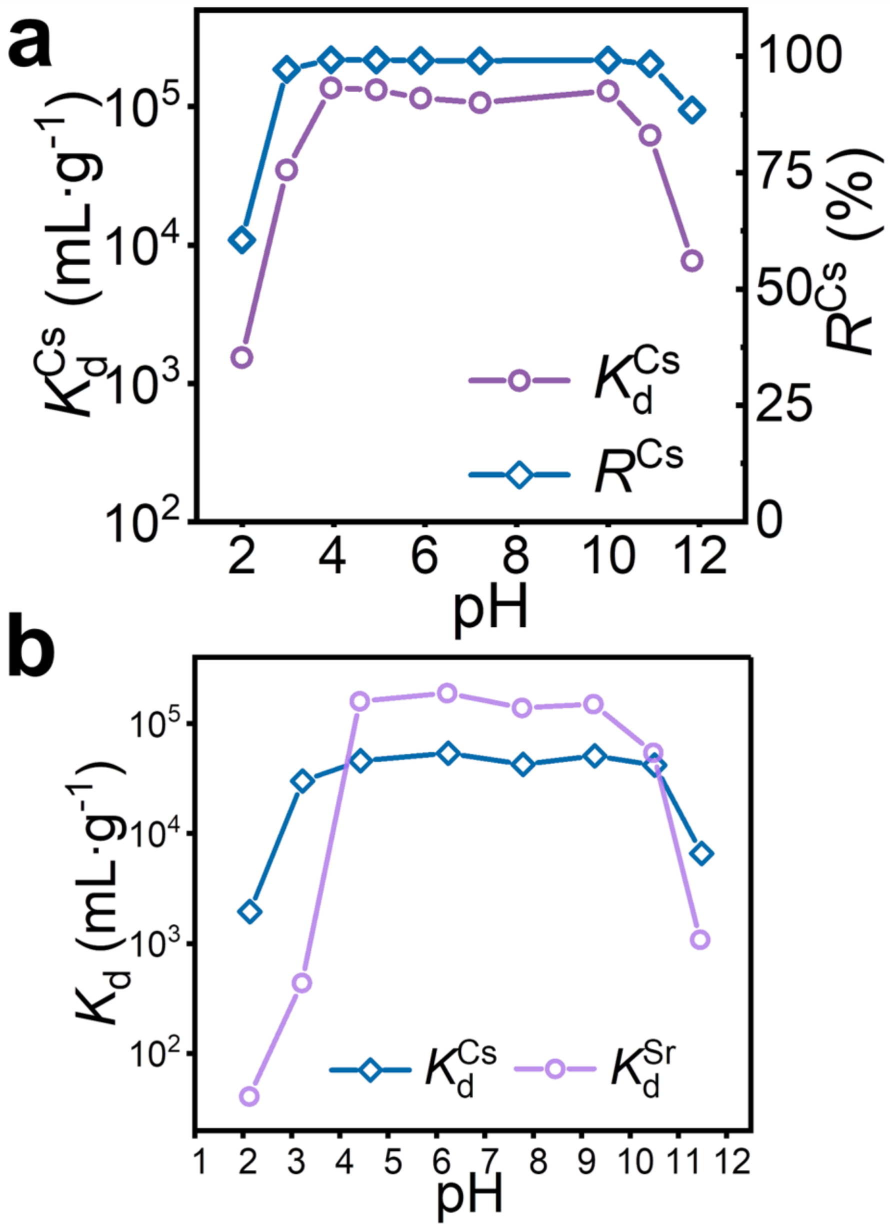

Supplementary Fig. 12. Effect of pH on the Cs^+ capture performance of FJSM-KCGTS. (a) K_d^{Cs} and R^{Cs} values of FJSM-KCGTS at various initial pH values. The purple and blue lines are artificially added to reflect the variation trend of the K_d^{Cs} and R^{Cs}, respectively. (b) K_d^{Cs} and K_d^{Sr} values of FJSM-KCGTS in solutions with the coexistence of Cs^+ and Sr^{2+} at various initial pH values. The purple and blue lines are artificially added to reflect the variation trend of the K_d^{Cs} and K_d^{Sr}, respectively. Source data are provided as a Source Data file.

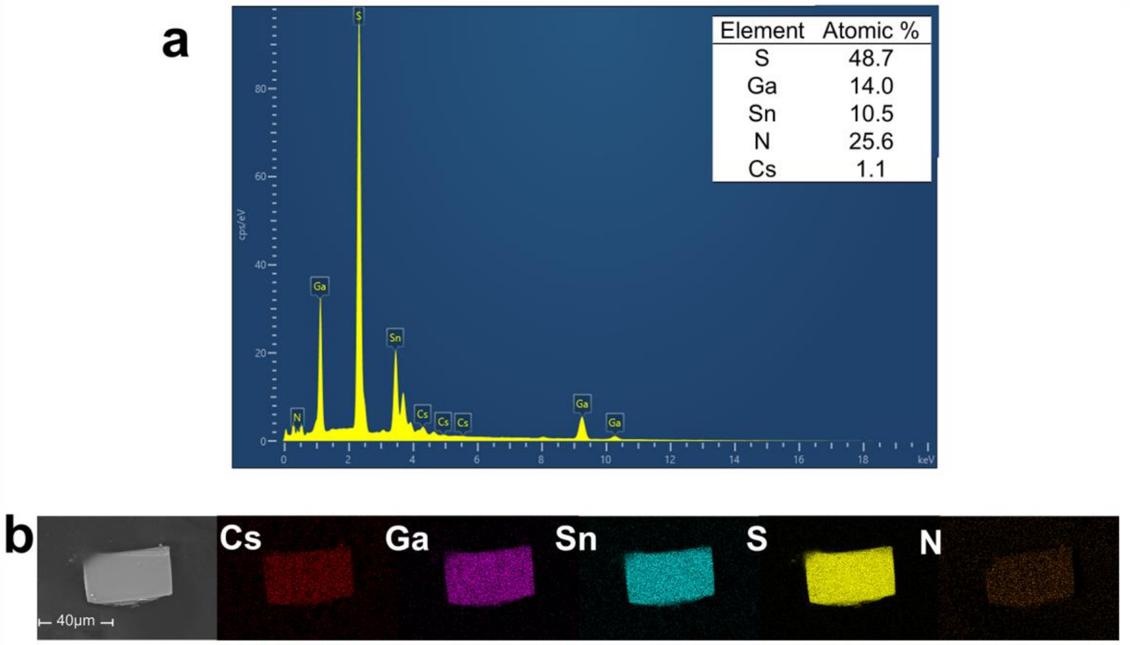

Supplementary Fig. 13. Elemental distribution of FJSM-KCGTS-Cs sample after elution. (a) EDS analysis results and (b) elemental distribution map of FJSM-KCGTS-Cs sample after elution with 1 mol·L^{-1} NH_4Cl solution.

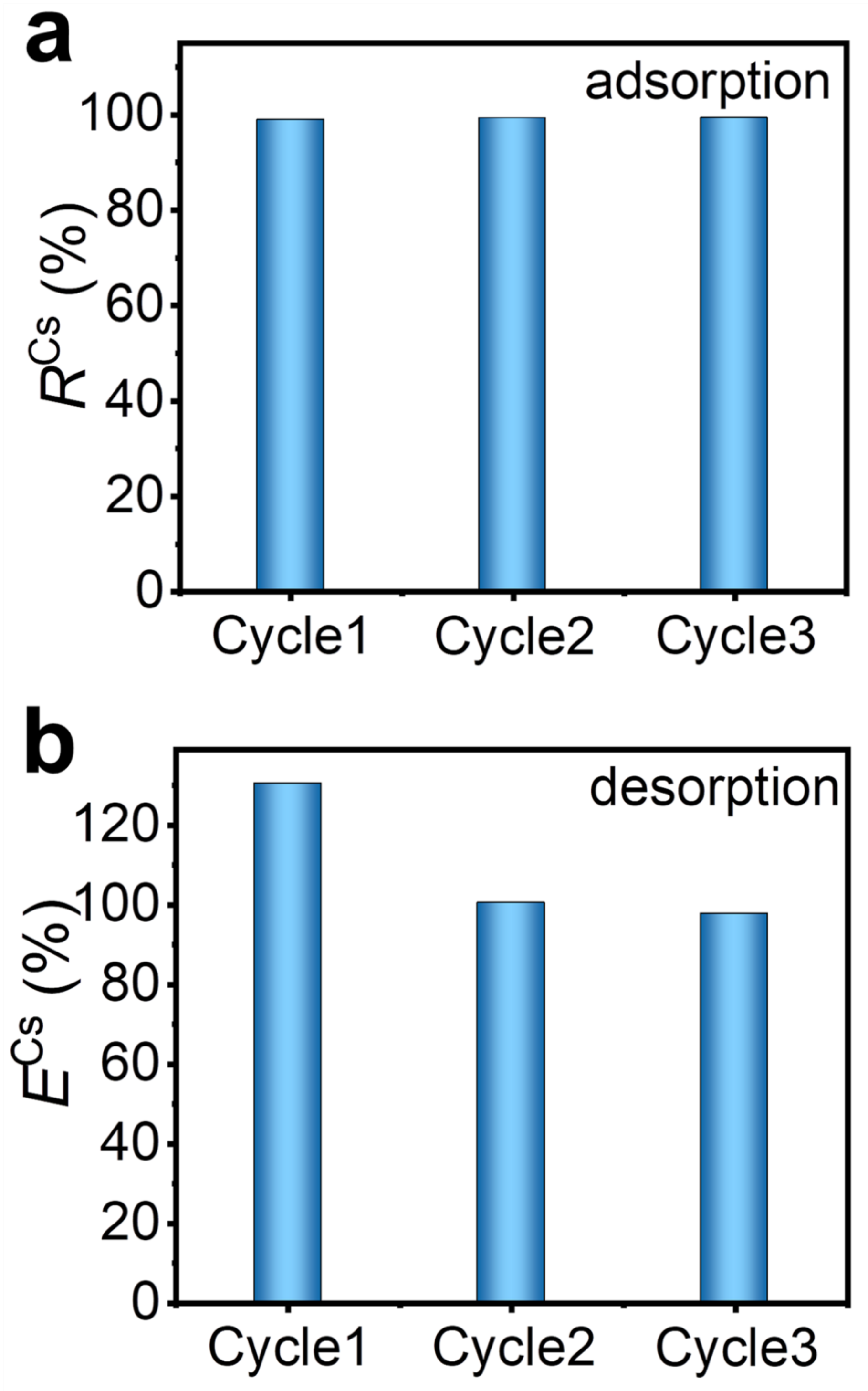

Supplementary Fig. 14. Recycling performance of FJSM-KCGTS. (a) Adsorption rates (R^{Cs}) and (b) desorption rates (E^{Cs}) at each cycle. Source data are provided as a Source Data file.

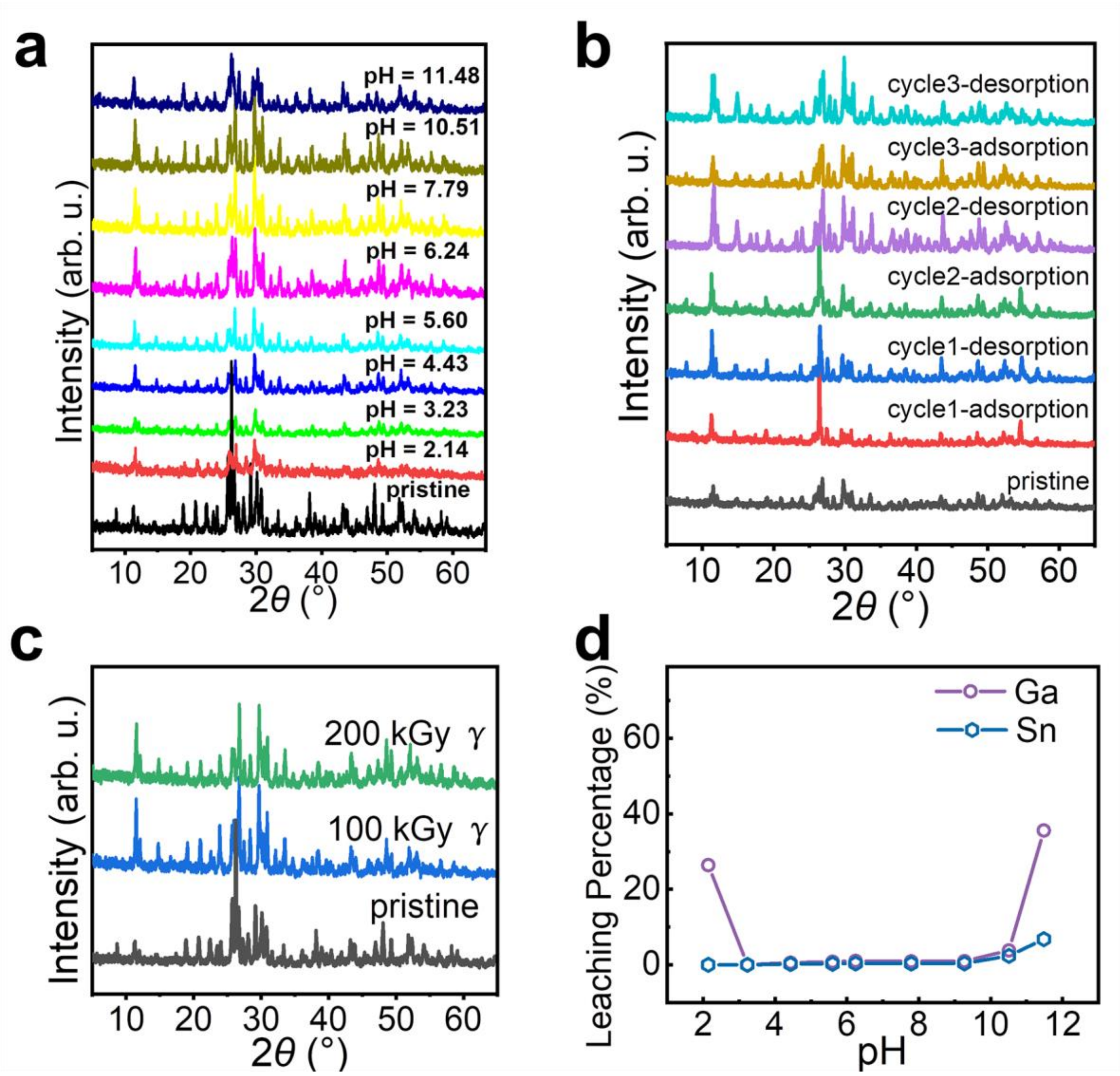

Supplementary Fig. 15. Stability study of FJSM-KCGTS. (a) PXRD patterns of exchange products in solutions with the coexistence of Cs^{+} and Sr^{2+} at various initial pH values. (b) PXRD patterns of samples after each round of adsorption and desorption. (c) PXRD pattern of FJSM-KCGTS samples before and after irradiation. (d) Leaching percentage of Ga^{3+} and Sn^{4+} from FJSM-KCGTS frameworks in solutions with the coexistence of Cs^{+} and Sr^{2+} at various initial pH values. The purple and blue lines are artificially added to reflect the variation trend of the leaching percentage of Ga^{3+} and Sn^{4+}, respectively. Source data are provided as a Source Data file.

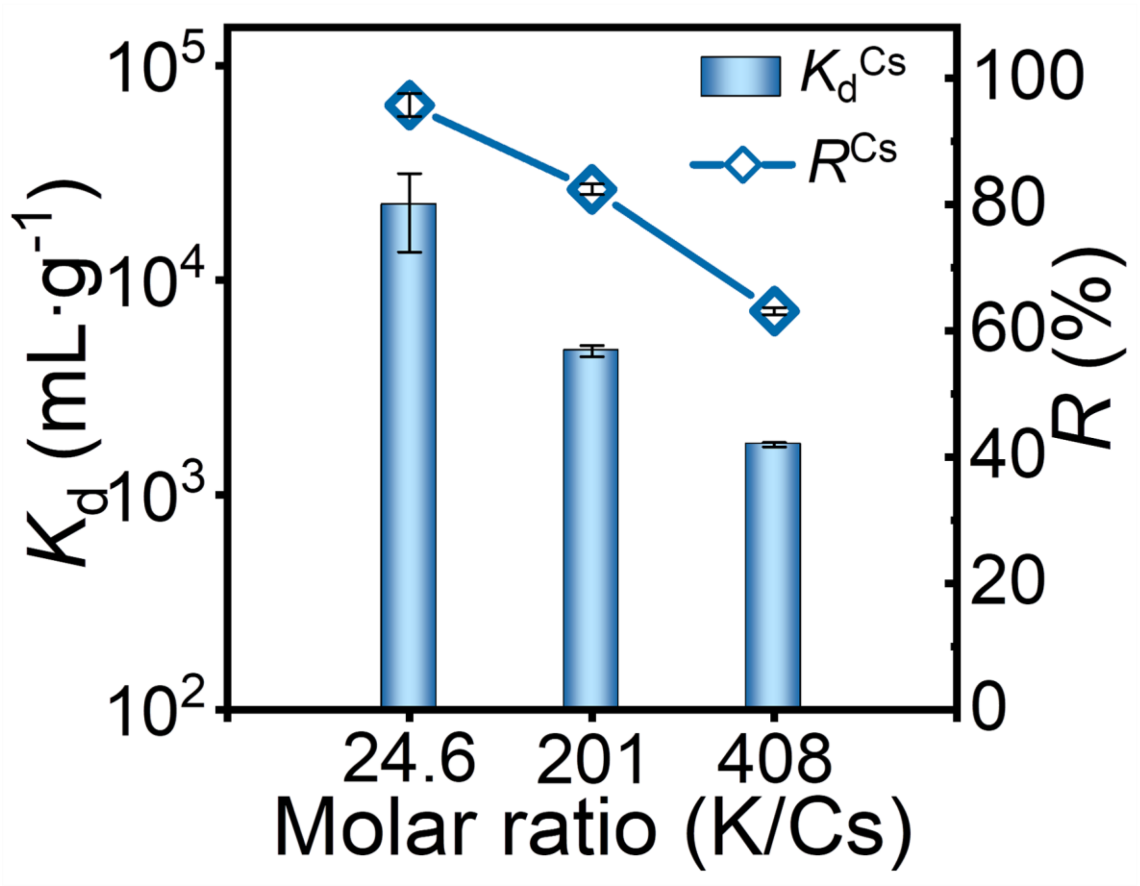

Supplementary Fig. 16. Effect of K^+ on selective capture of Cs^+ by FJSM-KCGTS. K_d^{Cs} and R^{Cs} values of FJSM-KCGTS in neutral solutions with different K/Cs molar ratios. The blue line is artificially added to reflect the variation trend of the R^{Cs}. Error bars present the standard deviation of the mean of three experiments. Source data are provided as a Source Data file.

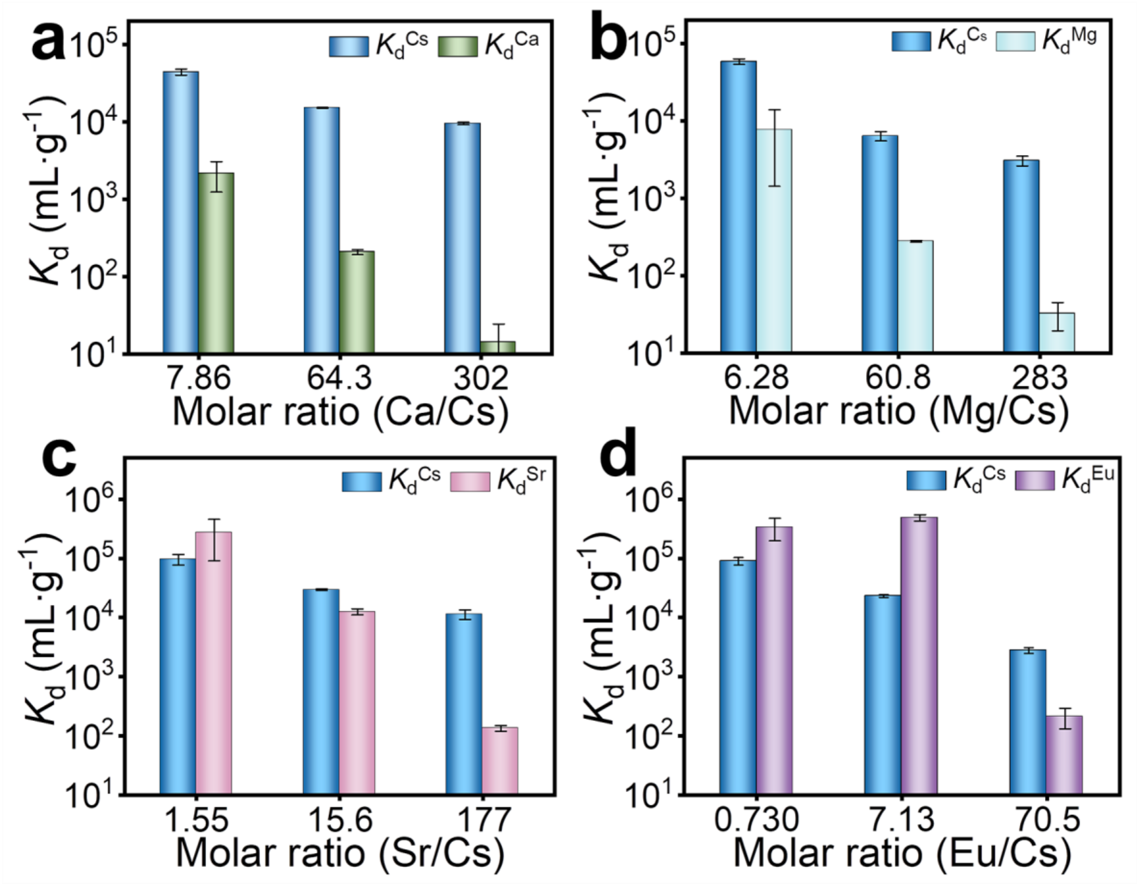

Supplementary Fig. 17. Effect of high-valency competing ions on selective capture of Cs^+ by FJSM-KCGTS. K_d of Cs^+ and M^{n+} (M^{n+} = Ca^{2+}, Mg^{2+}, Sr^{2+}, Eu^{3+}) ions removed by FJSM-KCGTS in neutral solutions with different (a) Ca/Cs, (b) Mg/Cs, (c) Sr/Cs, and (d) Eu/Cs molar ratios. Error bars present the standard deviation of the mean of three experiments. Source data are provided as a Source Data file.

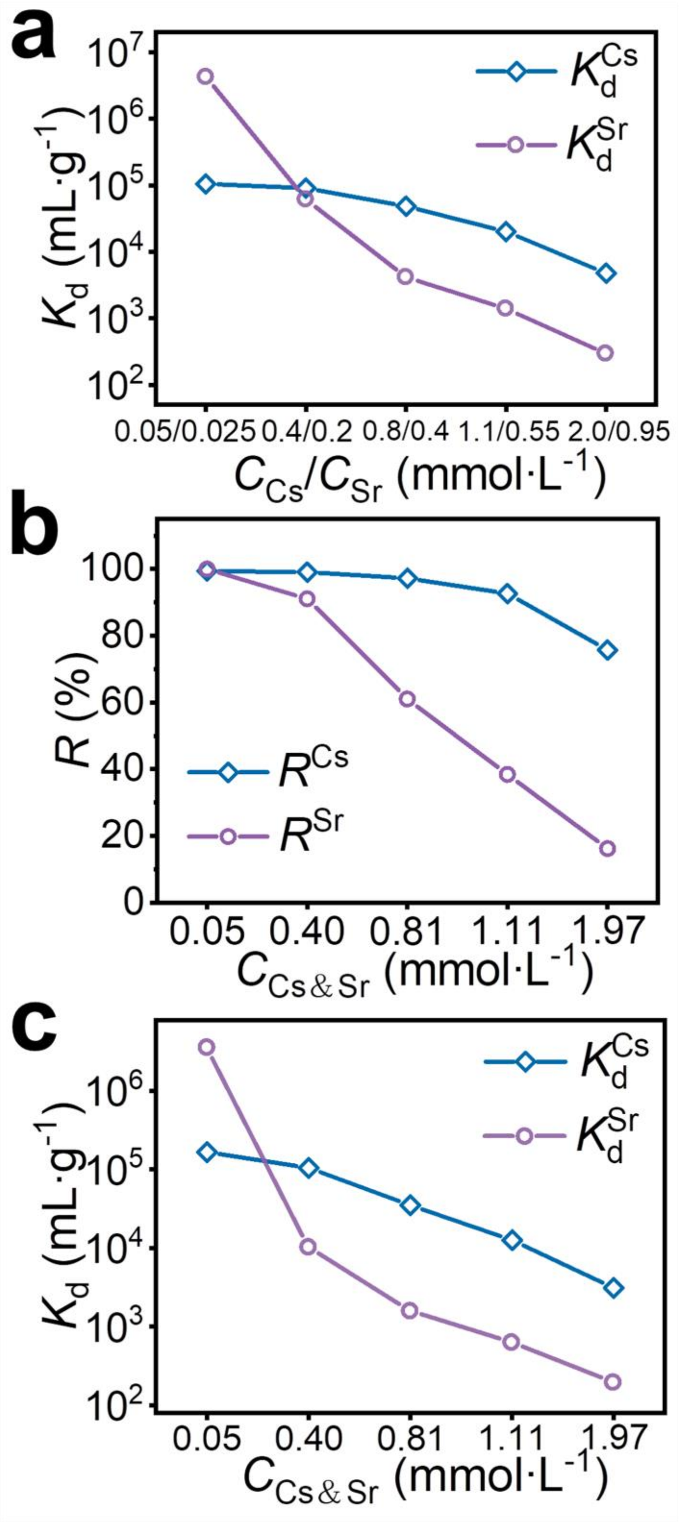

Supplementary Fig. 18. Effect of Sr^{2+} on selective capture of Cs^+ by FJSM-KCGTS. (a) of Cs^+ and Sr^{2+} ions removed by FJSM-KCGTS in neutral Sr/Cs solutions with equipotent charge concentration ($C_0^{Cs} = 2C_0^{Sr}$). (b) R and (c) K_d of Cs^+ and Sr^{2+} ions removed by FJSM-KCGTS in neutral solutions containing Cs^+ and Sr^{2+} ions with equimolar concentration ($C_0^{Cs} = C_0^{Sr}$). In Fig. 18a and 18c, the purple and blue lines are artificially added to reflect the variation trend of K_d^{Sr} and K_d^{Cs}, respectively. In Fig. 18b, the purple and blue lines are artificially added to reflect the variation trend of R^{Sr} and R^{Cs}, respectively. Source data are provided as a Source Data file.

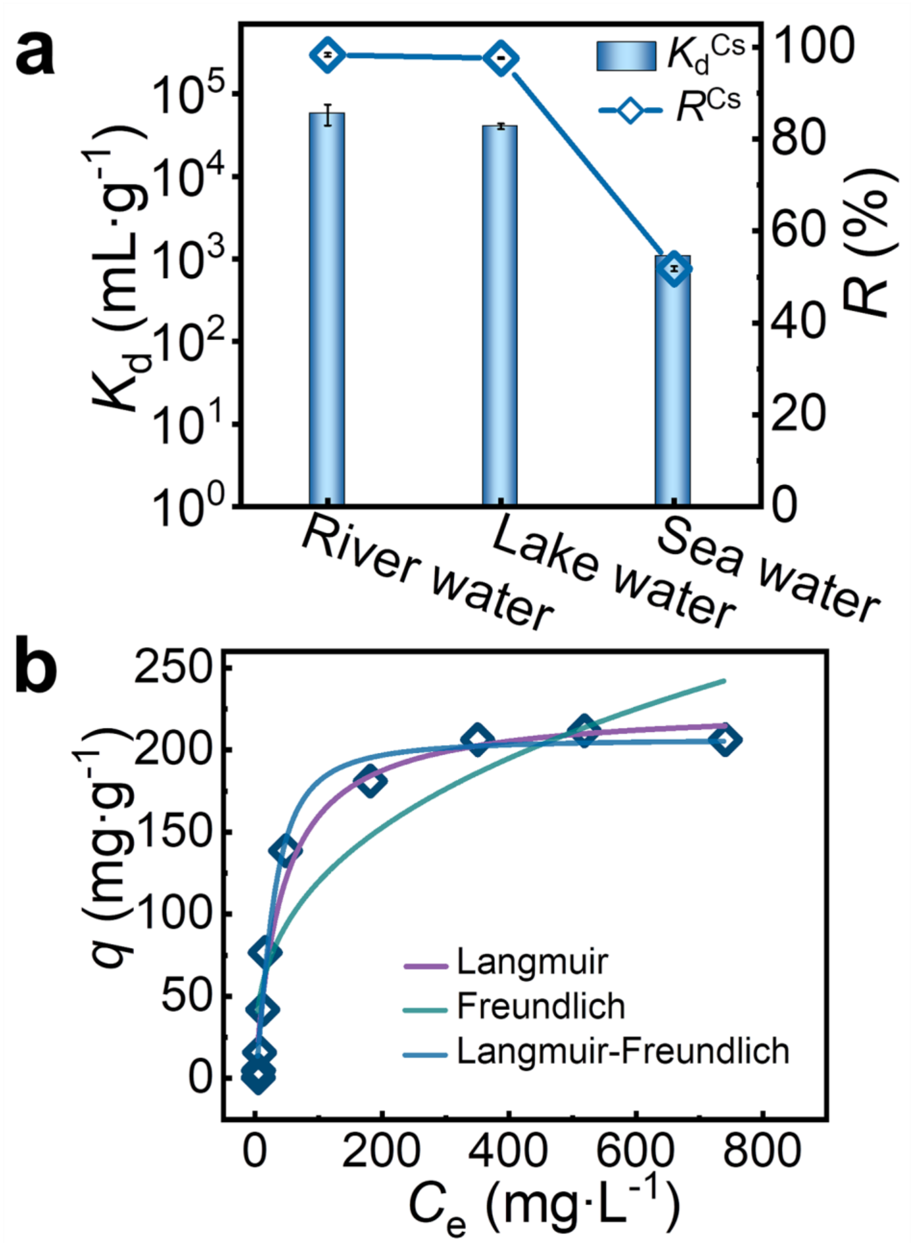

Supplementary Fig. 19. Performance of FJSM-KCGTS in capturing Cs^+ in simulated polluted environmental water samples. (a) K_d and R of Cs^+ ions captured by FJSM-KCGTS in simulated contaminated environmental water samples. The blue line is artificially added to reflect the variation trend of R^{Cs}. (b) Equilibrium data for Cs^+ ion exchange by FJSM-KCGTS in seawater with different initial concentrations of Cs^+ fitted with the Langmuir, Freundlich, and Langmuir-Freundlich isotherm models. Error bars present the standard deviation of the mean of three experiments. Source data are provided as a Source Data file.

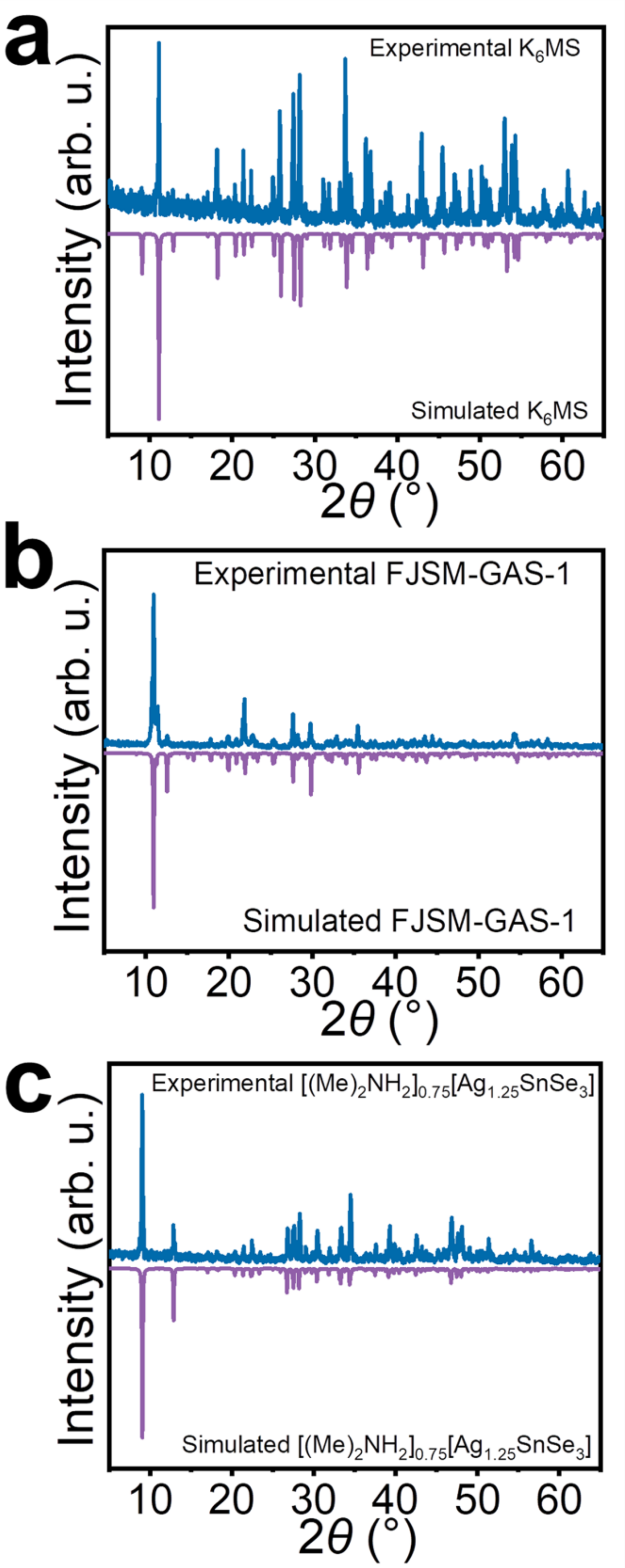

Supplementary Fig. 20. Pure phase validation of synthetic adsorbents. Experimental and simulated PXRD patterns of (a) 3D-K_6MS, (b) 2D-FJSM-GAS-1, and (c) 3D-$[(Me)_2NH_2]_{0.75}[Ag_{1.25}SnSe_3]$. Source data are provided as a Source Data file.

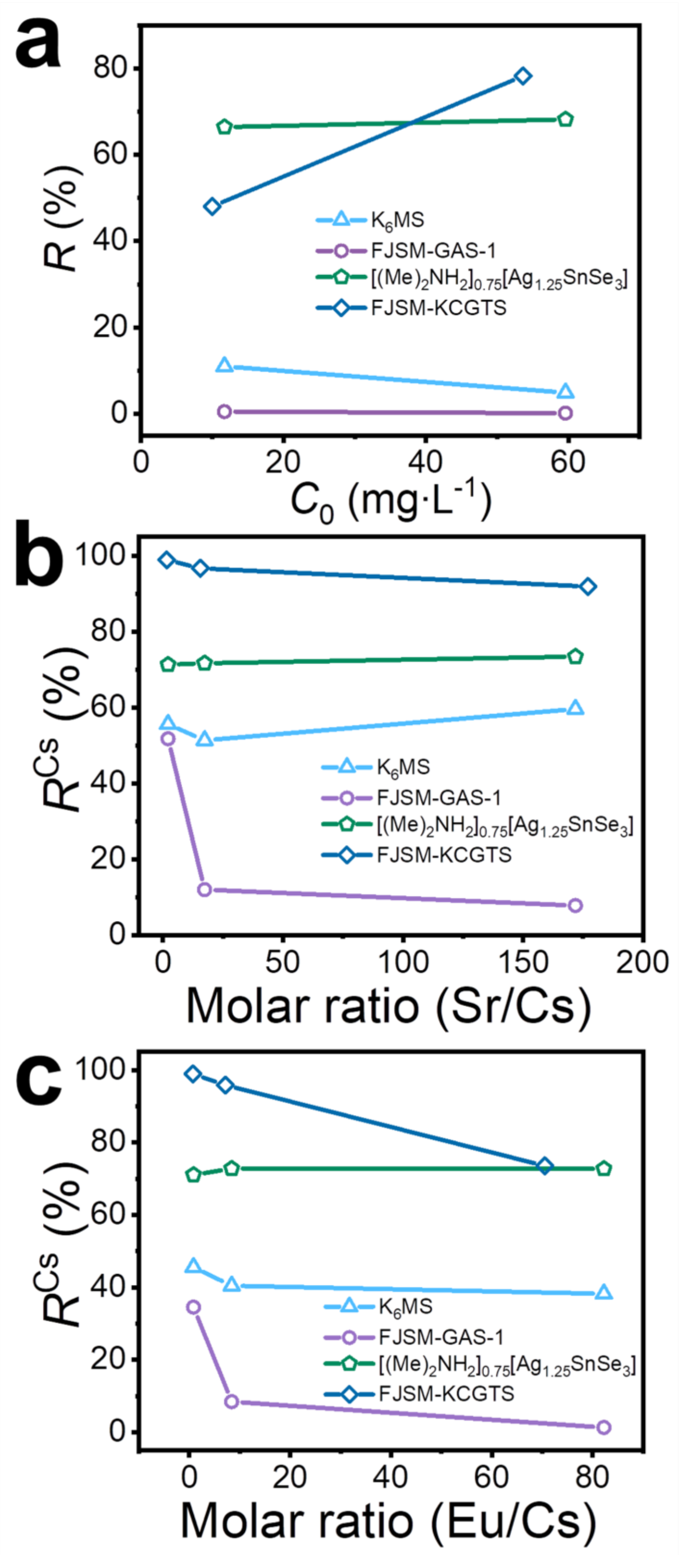

Supplementary Fig. 21. Comparison of Cs^+ selective capture capacity of different adsorbents. Comparison of Cs^+ removal by 3D-K_6MS, 2D-FJSM-GAS-1, 3D-$[(Me)_2NH_2]_{0.75}[Ag_{1.25}SnSe_3]$, and FJSM-KCGTS in (a) seawater samples, and in solutions with different (b) Sr/Cs and (c) Eu/Cs molar ratios. The lines in the figures are artificially added to reflect the variation trend of Cs^+ removal rates of the compounds. Source data are provided as a Source Data file.

Supplementary Table 8. Isotherm fitting parameters for Cs^+ capture by FJSM-KCGTS in seawater with different initial concentrations of Cs^+.

Langmuir model			
q_m (mg g^{-1})	b (L mg^{-1})	R^2	
226.94	0.02378	0.97436	
Freundlich model			
K_F [(mg g^{-1})(L mg^{-1})$^{1/n}$]	n	R^2	
23.256	2.8217	0.87099	
Langmuir-Freundlich model			
q_m (mg g^{-1})	b (L mg^{-1})	n	R^2
206.44	0.03559	0.6595	0.98827

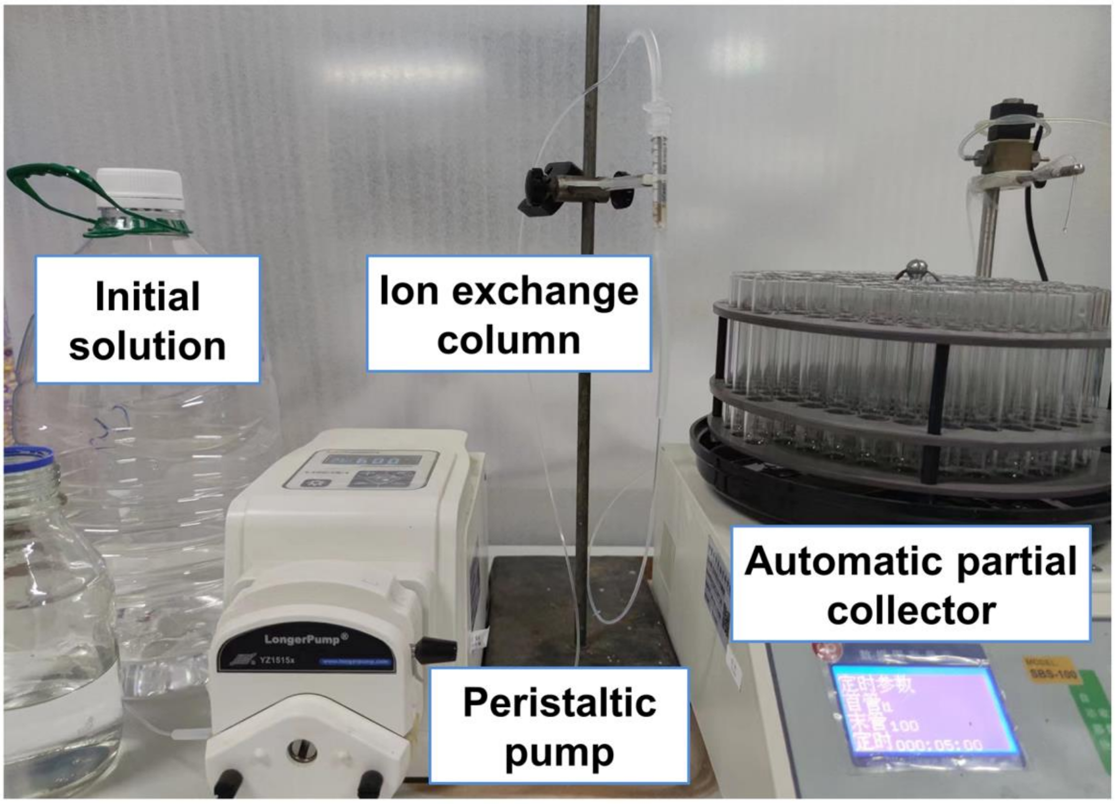

Supplementary Fig. 22. Experimental setup for ion exchange column experiments.

Supplementary Table 9. Thomas model fitting parameters for the experimental data in ion exchange column.

Solutions	K_T (L min^{-1} mg^{-1})	q_e (mg g^{-1})	R^2
31.995 mg L^{-1} Cs^+	1.53×10^{-4}	288.94	0.9957

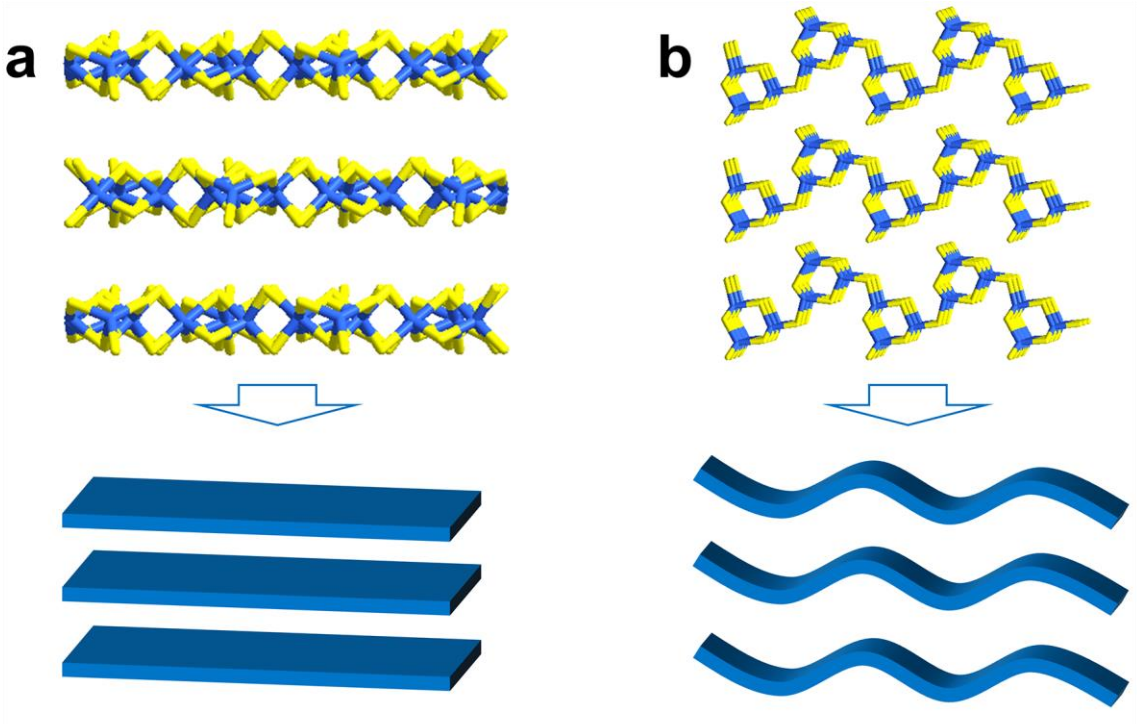

Supplementary Fig. 23. Schematic diagram of flat and wavy layer stacking. (a) Schematic diagram of plate-like layer stacking of FJSM-SnS (Blue ball: Sn, yellow ball: S). (b) Schematic diagram of wave-like layer stacking of FJSM-CGTS (Blue ball: Sn/Ga, yellow ball: S).

Supplementary Table 10. Layer spacing of metal sulfide ion exchangers after they capture Cs^+ or K^+ ions.

Compounds	Interlayer distance before ion-exchange		Interlayer distance after ion-exchange		Δ*d* (Å)	Ref.
	Ions in the interlayer	Layer spacing *d* (Å)	Ions in the interlayer	Layer spacing *d* (Å)		
FJSM-SnS	$[Me_2NH_2]^+$, $[Me_3NH]^+$	7.258	Cs^+	6.709	0.549	[10]
NIAS	NH_4^+	8.25	K^+	8.65	0.4	[11]
KIAS	K^+	8.65	Cs^+	8.25	0.4	
InSnS-1	K^+	8.431	Cs^+	8.69	0.259	[12]
KMS-1	K^+	8.47	Cs^+	8.96	0.49	[13]
KMS-2	K^+	8.52	Cs^+	8.99	0.47	[14]
KTS-3	K^+	8.441	Cs^+	8.813	0.372	[15]
**FJSM-CGTS**	**Cs^+**	**10.15**	**Cs^+, K^+**	**9.96**	**0.19**	**This work**
**FJSM-KCGTS**	**Cs^+, K^+**	**9.96**	**Cs^+, K^+**	**10.19**	**0.23**	

Supplementary References

1. Ho, Y. S., Wase, D. A. J. & Forster, C. F. Kinetic studies of competitive heavy metal adsorption by sphagnum Moss Peat. *Environ. Technol.* **17**, 71-77 (1996).

2. Duong, D. D. *Adsorption Analysis: Equilibria and Kinetics* (1998).

3. Mathialagan, T. & Viraraghavan, T. Adsorption of cadmium from aqueous solutions by perlite. *J. Hazard. Mater.* **94,** 291-303 (2002).

4. Chen, Z., Wu, Y., Wei, Y. & Mimura, H. Preparation of silica-based titanate adsorbents and application for strontium removal from radioactive contaminated wastewater. *J. Radioanal. Nucl. Chem.* **307,** 931-940 (2016).

5. Manos, M. J., Iyer, R. G., Quarez, E., Liao, J. H. & Kanatzidis, M. G. $\{Sn[Zn_4Sn_4S_{17}]\}^{6-}$: A robust open framework based on metal-linked penta-supertetrahedral $[Zn_4Sn_4S_{17}]^{10-}$ clusters with ion-exchange properties. *Angew. Chem., Int. Ed.* **44**, 3552-3555 (2005).

6. Manos, M. J. & Kanatzidis, M. G. Metal sulfide ion exchangers: superior sorbents for the capture of toxic and nuclear waste-related metal ions. *Chem. Sci.* **7**, 4804-4824 (2016).

7. Li, J. R. & Huang, X. Y. $[(Me)_2NH_2]_{0.75}[Ag_{1.25}SnSe_3]$: A three-dimensionally microporous chalcogenide exhibiting framework flexibility upon ion-exchange. *Dalton Trans.* **40,** 4387-4390 (2011).

8. Liu, H. W., et al. Deep eutectic solvothermal synthesis of an open framework copper selenidogermanate with pH-resistant Cs^+ ion exchange properties. *Chem. Commun.* **55,** 13884-13887 (2019).

9. Ding, D., et al. Efficient Cs^+-Sr^{2+} separation over a microporous silver selenidostannate synthesized in deep eutectic solvent. *Inorg. Chem.* **59,** 9638-9647 (2020).

10. Qi, X. H., et al. A Two-dimensionally microporous thiostannate with superior Cs^+ and Sr^{2+} ion-exchange property. *J. Mater. Chem. A* **3**, 5665-5673 (2015).

11. Zeng, X., et al. Ultra-fast 137Cs sequestration via a layered inorganic indium thioantimonate. *Environ. Sci.: Adv.* **1**, 331-341 (2022).

12. Tang, J. H., et al. Highly selective cesium(I) capture under acidic conditions by a layered sulfide. *Nat. Commun.* **13**, 658 (2022).

13. Manos, M. J. & Kanatzidis, M. G. Highly efficient and rapid Cs^+ uptake by the layered metal sulfide $K_{2x}Mn_xSn_{3-x}S_6$ (KMS-1). *J. Am. Chem. Soc.* **131**, 6599-6607 (2009).

14. Mertz, J. L., Fard, Z. H., Malliakas, C. D., Manos, M. J. & Kanatzidis, M. G. Selective removal of Cs^+, Sr^{2+}, and Ni^{2+} by $K_{2x}Mg_xSn_{3-x}S_6$ (x = 0.5~1) (KMS-2) relevant to nuclear waste remediation. *Chem. Mater.* **25**, 2116-2127 (2013).

15. Sarma, D., Malliakas, C. D., Subrahmanyam, K. S., Islama, S. M. & Kanatzidis, M. G. $K_{2x}Sn_{4-x}S_{8-x}$ (x = 0.65~1): a new metal sulfide for rapid and selective removal of Cs^+, Sr^{2+} and UO_2^{2+} ions. *Chem. Sci.* **7**, 1121-1132 (2016).
